# Governance-aware autonomous retail coordination in artificial intelligence cities using multi-agent reinforcement learning, blockchain accountability, and federated learning

**DOI:** 10.3389/frai.2026.1942376

**Published:** 2026-10-07

**Authors:** Toqeer Ali Syed, Ali Akarma, Shahid Kamal, Salman Jan, Ahmad B. Alkhodre, Arshad Jamal

**Affiliations:** 1 AI Center, Faculty of Computer and Information Systems, Islamic University of Madinah, Madinah, Saudi Arabia; 2 AI V&V Lab, King Fahd University of Petroleum and Minerals, Dhahran, Saudi Arabia; 3 Center for Advanced Analytics, CoE for Artificial Intelligence, Faculty of Computing & Informatics, Multimedia University, Cyberjaya, Selangor, Malaysia; 4 Faculty of Computer Studies, Arab Open University-Bahrain, A'Ali, Bahrain

**Keywords:** utonomous inventory replenishment, blockchain accountability, federated learning, future of work, human oversight, multi-agent reinforcement learning, sustainable cities, urban AI governance

## Abstract

When autonomous systems take operational authority over urban commerce, accountability and human oversight matter as much as efficiency. We present AAIRM, a governance-aware autonomous retail coordination framework addressing three requirements for trustworthy procurement: tamper-evident decision provenance, data sovereignty, and adaptive demand coordination. AAIRM couples a Proximal Policy Optimization (PPO) ordering policy, a permissioned Blockchain Trust Ledger (BTL) for multi-party audit trails, and a Federated Demand Learning (FDL) layer. Evaluation is simulation-based, using a multi-category synthetic environment and the public M5 dataset; no live retail deployment is claimed. At its cost-optimal operating point (92.3% fill), AAIRM lowers normalized inventory cost by 13.2% (95% CI 12.2–14.2) against a conventionally parameterized reorder-point/economic-order-quantity (ROP–EOQ) baseline and by 10.2% (95% CI 8.0–12.4) on M5. Service parity is obtained by moving AAIRM along its own cost–service frontier rather than by retuning baselines: At 96.1% and 97.4% fill, it retains 8.2-point and 4.9-point advantages. A layered ablation attributes 8.8 points of the advantage to the reinforcement learning (RL) policy and 3.8 points to the combined coordination-and-governance block, which we further decompose into feasibility projection, escalation logic, cross-category policy rules, supplier ranking, and negotiation. The language-model orchestrator contributes 0.6 points (95% CI −0.9 to 2.1), statistically indistinguishable from zero and practically equivalent within a ±2-point margin. Against a feasibility-matched multi-agent RL comparator, the residual cost gap does not survive multiple-comparison correction, so the defensible advantages are hard feasibility and auditability, not cost. The BTL adds 6.6% decision-cycle overhead; its 500-case mutation replay is an integration check, and we give an explicit adversary model showing that crash-fault-tolerant ordering does not resist colluding majorities. FedProx converges within 0.9 WAPE points of centralized training at a 1.3-point cost penalty, without secure aggregation or differential privacy; we analyze rather than evaluate when those mechanisms become necessary. A 500-SKU study exposes a reward-hacking failure in a high-spoilage category, corrected by category-specific governance. We close with an architectural, non-empirical reading of labor exposure and three governance instruments regulators can act on.

## 1 Introduction

Autonomous systems now manage critical urban infrastructure: energy grids, transportation networks, and, with growing consequence, the retail supply chains that stock stores and distribution centers throughout modern cities. Retail in this framing is a distributed urban service system as much as a commercial sector, and its reliability bears directly on food security, consumer welfare, and city-level economic resilience (IHL Group, 2023). As artificial intelligence (AI) systems take decision authority over procurement and replenishment at city scale, the governance questions that have animated debates about autonomous vehicles and algorithmic hiring apply with equal force to the agents ordering goods on behalf of millions of residents. The transition is underway: against an estimated $1.77 trillion global cost of inventory inefficiencies (IHL Group, 2023; Marjerison et al., 2025), a 2020 benchmarking study of 104 global retailers found that only 15% had reached prescriptive or autonomous supply chains, with 22% still planning demand on spreadsheets (Blue Yonder and WMG, University of Warwick). Who fills that gap, under whose oversight, and which urban retail roles are reshaped in the process are questions of political economy as much as of operations.

Three structural barriers separate current practice from trustworthy autonomous replenishment. The first is adaptivity. Classical models such as economic order quantity (EOQ) and reorder point (ROP) rely on offline demand parameters that are refreshed infrequently (Lee et al., 2015). Attaching a machine-learning forecast to unchanged rule-based order logic can even raise cost by overstocking slow-moving categories, and our BL2 baseline (Section 4.2) reproduces that pattern as a 13.2% cost increase over ROP–EOQ. The second is accountability. When agents procure without continuous human oversight, the audit trail has to be independent of the system it monitors. A centralized log that a compromised orchestrator or a privileged insider can alter cannot supply verifiable decision provenance (Saberi et al., 2019; Kshetri, 2018; Cole et al., 2019). The third is data sovereignty. Competitive, contractual, and privacy constraints keep multi-store networks from pooling sales data; however, collaborative demand modeling improves accuracy substantially. Federated learning resolves that tension in principle by training a shared model without moving raw observations (McMahan et al., 2017; Kairouz et al., 2021).

These barriers converge on one systemic problem: No existing framework provides an end-to-end autonomous procurement pipeline that is simultaneously cost-efficient, accountable through tamper-evident auditing, and privacy-preserving under data-sovereignty constraints. Accountable Autonomous Inventory Replenishment and Management (AAIRM) addresses all three in a unified multi-agent architecture. Our own findings led us to frame the contribution around the governance and trust subsystems rather than Large Language Model (LLM)-mediated reasoning. Those subsystems supply hard feasibility and auditability that strong multi-agent reinforcement learning (RL) does not, whereas the language model adds no statistically significant value in structured procurement. We report results at the service levels a grocery retailer would run in practice (95%–98% fill) and note the leaner operating point separately.

### 1.1 Scope and evidentiary status of the claims

Because this study spans operations research, distributed systems, and governance, we state at the outset what each claim rests on. Four evidentiary tiers run through the study, and we keep them separate throughout. Operational results at 100 and 500 stock keeping units (SKUs) come from a controlled multi-category simulator. They are internally valid and reproducible from the released code, but they remain simulation results rather than field measurements. External validity is probed on a single public corpus, the M5 Forecasting Competition data, which covers one retailer in one country. Ledger results measure a four-organization permissioned testbed under replayed load. The tamper-detection figure is an integration check that follows from cryptographic hash properties, not an adversarial security evaluation. The labor discussion in Section 5.4 is an architectural reading of task exposure, resting on no human subjects, no workforce data, and no measured employment outcome. Where the text says “urban” or “AI city,” it names the setting the architecture targets, not one in which the system has been observed to run.

Section 2 covers background and related work. Section 3 presents the architecture, including the governance specification, the trust ledger with its threat model, and the federated learning layer with its privacy model. Section 4 gives the experimental protocol and results, and Section 5 takes up governance implications, socio-technical risks, and limitations. Section 6 concludes and sets out the study's contributions.

## 2 Background and related work

Five technical pillars support the framework: classical inventory theory, AI and reinforcement learning for supply chain management, agentic systems, blockchain-based accountability, and federated learning. A sixth literature, on governance and algorithmic management, frames what accountable autonomy over urban infrastructure requires. Table 1 summarizes the positioning of this study relative to representative prior contributions.

**Table 1:** Positioning of the proposed AAIRM framework relative to representative prior work.

Work	Multi- category	Agentic coordination	RL policy	Hard feasibility	Supplier negotiation	Blockchain audit	Federated learning
Nahmias (1982)	°	—	—	—	—	—	—
Shen and Norrie (1999)	°	✓	—	—	—	—	—
Oroojlooyjadid et al. (2022)	—	—	✓	—	—	—	—
Gijsbrechts et al. (2022)	°	—	✓	—	—	—	—
Mousa et al. (2024)	°	°	✓	—	—	—	—
Achiam et al. (2017)	—	—	✓	°	—	—	—
Jannelli et al. (2025)	—	✓	—	—	✓	—	—
Quan and Liu (2024)	°	✓	—	—	—	—	—
Saberi et al. (2019)	—	—	—	—	—	✓	—
Kshetri (2018)	—	—	—	—	—	✓	—
McMahan et al. (2017)	—	—	—	—	—	—	✓
**AAIRM (this work)**	✓	✓	✓	✓	✓	✓	✓

✓indicates full support; ° indicates partial support; —indicates the property is absent. “Hard feasibility” denotes a mechanism that guarantees joint budget and capacity satisfaction by construction rather than through a learned penalty. Bold entries indicate the results corresponding to the proposed work (AAIRM framework).

### 2.1 Inventory models, perishability, and multi-category coordination

Classical inventory theory sizes stocking and replenishment decisions through well-established formulas: EOQ, safety stock, lead-time buffering, and the reorder point (Nahmias and Olsen, 2020). The canonical ROP formulation is


$$\begin{array}{l} \text{ROP}=\mu_{D}\cdot L+z\cdot \sigma_{D}\sqrt{L}, \end{array}$$ (1)

where *L* is the replenishment lead time, *z* is the safety factor for the target service level, and μ_*D*_ and σ_*D*_ are the mean and standard deviation of per-period demand. Equation 1 requires accurate, stationary estimates of μ_*D*_ and σ_*D*_, an assumption that rarely holds in multi-category retail. Modern supply chains are networked rather than single-echelon (Axsäter, 2015), and this structure produces the bullwhip effect, in which small demand fluctuations amplify upstream into costly production and inventory oscillations (Lee et al., 2015). Perishable and category-diverse assortments add expiration modeling and cross-category priority structures, from the foundational analytical models (Nahmias, 1982) through stochastic multi-period extensions (Karaesmen et al., 2011) to Internet-of-Things freshness tracking (Weißhuhn and Hoberg, 2021). Collaborative Planning, Forecasting and Replenishment and vendor-managed inventory dampen bullwhip instability in part, but both depend on manual parameter adjustment (Sari, 2008). For AI-driven supply chains, Kumar et al. (2023) identify data integration, stakeholder buy-in, and governance as the critical success factors.

### 2.2 AI and reinforcement learning for lnventory and procurement

AI has reshaped demand forecasting, procurement, and replenishment across supply chain research. A review of 803 papers found forecasting, planning, and decision support to be the most common applications (Toorajipour et al., 2021). Machine-learning forecasting consistently beats rule-based baselines on responsiveness and scalability, although data integration, interoperability, and interpretability remain deployment barriers (Culot et al., 2024). Coupling machine learning with optimization reduces stockouts and holding costs by updating reorder criteria in real time (Yeldan et al., 2024). Douaioui et al. (2024) called for deeper integration between demand-forecasting architectures, now dominated by Long Short-Term Memory (LSTM) and Transformer models, and the downstream inventory decisions those forecasts feed. AAIRM addresses that gap directly.

Reinforcement learning has attracted particular attention for adaptive ordering. Boute et al. (2022) charted its design challenges in inventory control: state representation, reward shaping, and multi-echelon coordination. A Deep Q-Network (DQN) on the beer game outperformed classical base-stock policies and cut the bullwhip effect substantially (Oroojlooyjadid et al., 2022), and deep RL matches or exceeds optimal heuristics on lost-sales, dual-sourcing, and multi-echelon problems (Gijsbrechts et al., 2022). Later work extended RL to structured multi-echelon settings (Geevers et al., 2024; Stranieri et al., 2024), applied PPO to stochastic capacitated lot-sizing (van Hezewijk et al., 2023), studied multi-agent RL for decentralized control and demand-supply synchronization (Mousa et al., 2024; Kegenbekov and Jackson, 2021), and addressed perishables through reward shaping and distributional RL (De Moor et al., 2022; Wu et al., 2023). The lot-sizing result directly supports our choice of PPO for the Reorder Optimization Agent. These contributions share a focus on individual techniques in largely centralized settings. None provides the inter-agent governance, auditability, and data-sovereignty mechanisms that characterize the present study.

### 2.3 Constrained and safe reinforcement learning

Enforcing operational constraints on a learned policy is well studied, and the design space matters for interpreting our governance layer. Constrained Markov decision processes (Altman, 1999) formalize the setting. Lagrangian methods learn multipliers alongside the policy: Constrained Policy Optimization (Achiam et al., 2017) gives monotonic improvement guarantees under expected-cost constraints, PID-controlled multipliers damp the oscillation these methods commonly exhibit (Stooke et al., 2020), and standardized benchmarks are now available for the family (Ray et al., 2019). A complementary line enforces constraints structurally rather than through the objective, projecting or correcting actions at execution time so that violations cannot occur at all (Dalal et al., 2018). The distinction is consequential for procurement: Lagrangian formulations bound constraint violation in expectation, whereas a retailer's purchasing budget and warehouse volume are hard per-period ceilings that cannot be exceeded on any single cycle. AAIRM therefore adopts structural enforcement, and Section 4.7.1 discusses the trade-offs this choice entails. The reward-hacking failure documented in Section 4.9 connects to the broader alignment literature on objective misspecification (Amodei et al., 2016; Skalse et al., 2022; Pan et al., 2022; Jan et al., 2026), which shows that agents reliably exploit gaps between a stated objective and the intent behind it.

### 2.4 Agentic systems and autonomous supply chain frameworks

The most active current frontier is agentic AI: autonomous, reasoning-capable agents that perceive complex environments, form plans, and act across distributed supply chains. Surveys identify perception, planning, action, and memory as the essential modules of LLM-based agents (Wang et al., 2024) and document emergent collaboration in multi-agent LLM systems (Guo et al., 2024), while ReAct (Yao et al., 2023) established interleaved reasoning and tool action as the foundational pattern. Earlier multi-agent system research showed that local decision autonomy reduces global coordination costs (Shen and Norrie, 1999) and demonstrated the benefits of distributed decision-making under uncertainty (Swaminathan et al., 1998). Related work addressed multi-agent scheduling with resource allocation (Aminzadegan et al., 2019) and the role of AI in redefining purchasing (Allal-Chérif et al., 2021). In the LLM-agent domain, Jannelli et al. (2025) proposed autonomous negotiation in place of dynamic optimization, Quan and Liu (2024) proposed the InvAgent multi-agent inventory framework, Holland and Chaudhari (2024) applied LLM agents to manufacturing planning, and Xu et al. (2024) identified integration, trust, and governance as the critical barriers. Yu et al. (2022) provide the algorithmic foundations for the multi-agent PPO (MAPPO) coordination on which our comparator builds.

We situate the present framework explicitly against our earlier agentic replenishment work (Syed et al., 2025), which established the forecasting, stock-optimization, and adaptive-coordination pipeline that AAIRM inherits. The present study extends that foundation along five axes: the trust ledger with its adversary model, the federated layer with its privacy boundary, the reward-hacking analysis with its remedy, the LLM null result, and the strengthened baseline suite with external validation. The contribution here is therefore the governance and accountability layer and its empirical characterization, not the agentic replenishment loop itself.

### 2.5 Blockchain for supply chain accountability

Blockchain is an append-only, replicated, cryptographically chained transaction log whose integrity rests on consensus among independent parties rather than a single database administrator (Jan et al., 2025). Its most immediately attainable supply chain benefits are provenance, auditability, and tamper-evident records (Kshetri, 2018; Cole et al., 2019), with permissioned deployments as the realistic enterprise pathway (Saberi et al., 2019). Hyperledger Fabric (Androulaki et al., 2018; Syed et al., 2026a) offers identity-based membership, pluggable consensus, and smart-contract execution at throughput well above that of public proof-of-work chains. The property directly relevant to autonomous procurement is tamper evidence: No party, including the orchestrator that generated a decision, can silently modify the corresponding record, a guarantee centralized application logs cannot provide. Tamper evidence is not, however, the same as tamper resistance, and the distinction turns on the consensus layer. Fabric's default Raft ordering service (Ongaro and Ousterhout, 2014) tolerates crash failures but not Byzantine behavior, so guarantees against colluding participants require a Byzantine-fault-tolerant ordering service (Castro and Liskov, 1999; Barger et al., 2021). Section 3.4.1 makes the resulting boundary explicit. This literature treats blockchain primarily as a provenance layer for physical goods. Its use as an accountability substrate for autonomous procurement *agents*, anchoring machine decisions rather than shipment events, remains largely unexplored, and prior agentic frameworks (Jannelli et al., 2025; Quan and Liu, 2024; Xu et al., 2024) identify trust as a barrier without empirically characterizing a ledger-based solution.

### 2.6 Federated learning and its privacy boundary

Federated learning (FL) trains a shared model across decentralized data holders without transferring raw data (McMahan et al., 2017). In the canonical federated averaging (FedAvg) protocol, each client transmits only parameters obtained from local gradient steps, which a coordinator aggregates by weighted averaging. Statistical heterogeneity, systems heterogeneity, and privacy are the principal open challenges (Kairouz et al., 2021; Li et al., 2020a), and FedProx (Li et al., 2020b) adds a proximal term that promotes convergence under client heterogeneity. Withholding raw data is a meaningful but partial privacy guarantee. Parameter updates leak information about the data that produced them, as gradient-inversion (Zhu et al., 2019; Geiping et al., 2020) and membership-inference (Shokri et al., 2017; Syed et al., 2026b) attacks demonstrate, and malicious clients can poison the global model outright (Bagdasaryan et al., 2020). Secure aggregation (Bonawitz et al., 2017) ensures the coordinator observes only the aggregated update, differential privacy (Abadi et al., 2016; McMahan et al., 2018) supplies a formal bound at a measurable accuracy cost, and robust aggregation rules such as coordinate-wise median or Krum (Blanchard et al., 2017) address the poisoning adversary. Stores are natural FL clients in multi-store retail. Point-of-sale history stays local, and the shared model benefits from cross-store diversity in category mix and seasonality, a distribution that is not independent and identically distributed (non-IID) by nature. To our knowledge, no prior agentic inventory framework integrates federated demand training into the replenishment pipeline, quantifies its downstream inventory-cost gap against centralized and local-only training, and states the residual privacy exposure explicitly.

### 2.7 AI governance, algorithmic management, and urban autonomous systems

AI governance has become a critical research priority as autonomy expands from controlled industrial environments to city-scale infrastructure. Trust and governance are the central barriers to deploying foundation-model-enabled supply chains (Xu et al., 2024), and they are the barriers that AAIRM's ledger and Governance Agent address. Emerging regulation (European Parliament and Council of the European Union, 2024) imposes auditability, transparency, and human-oversight requirements on consequential procurement decisions, and verification, transparency, and accountability are the pillars of effective governance at scale (Dafoe, 2018). A closely related literature examines algorithmic management of human workers rather than autonomous procurement. Parent-Rocheleau and Parker (2022) review the monitoring, goal-setting, scheduling, and compensation functions that algorithms increasingly take over from human managers. Doellgast et al. (2023) document how worker representatives in Germany and Norway adapted collective-voice institutions to regulate algorithmic monitoring, a response pattern that bears directly on the escalation and ledger-permissioning instruments of Section 5.4. Muldoon et al. (2023) add that ethical claims made at the platform level do not guarantee equitable outcomes for the workers whose labor underlies the system. The wider critical-automation literature spans labor economics, where the displacement-vs.-augmentation debate remains unsettled (Frey and Osborne, 2017; Autor, 2015; Brynjolfsson and McAfee, 2014; Acemoglu and Restrepo, 2019), and critical AI studies, which foreground the distributional and extractive dimensions of automated decision systems (Eubanks, 2018; Crawford, 2021). Agentic supply chain frameworks engage this literature sparingly, treating governance as a technical reliability property rather than a socio-technical one. Our position is deliberately narrow. Accountability infrastructure is a precondition for engaging distributional questions at all because contesting how authority has been delegated requires first being able to observe and verify the decisions made under it.

## 3 Proposed framework

AAIRM implements inventory replenishment, procurement, and product discovery through a structured Perception–Conceptualization–Action workflow, illustrated in Figure 1. A central Meta-Orchestrator (LangGraph/LangChain) coordinates task decomposition, agent memory, and tool routing across all layers. A Federated Demand Learning layer connects per-store training clients to the Demand Forecasting Agent. An External Commerce Ecosystem of enterprise resource planning, warehouse management, supplier, logistics, and marketplace interfaces supplies the channels through which agents perceive and act on the retail environment. Principal notation is tabulated in Supplementary Table S1, which distinguishes the three symbols most easily confused.

**Figure 1:**
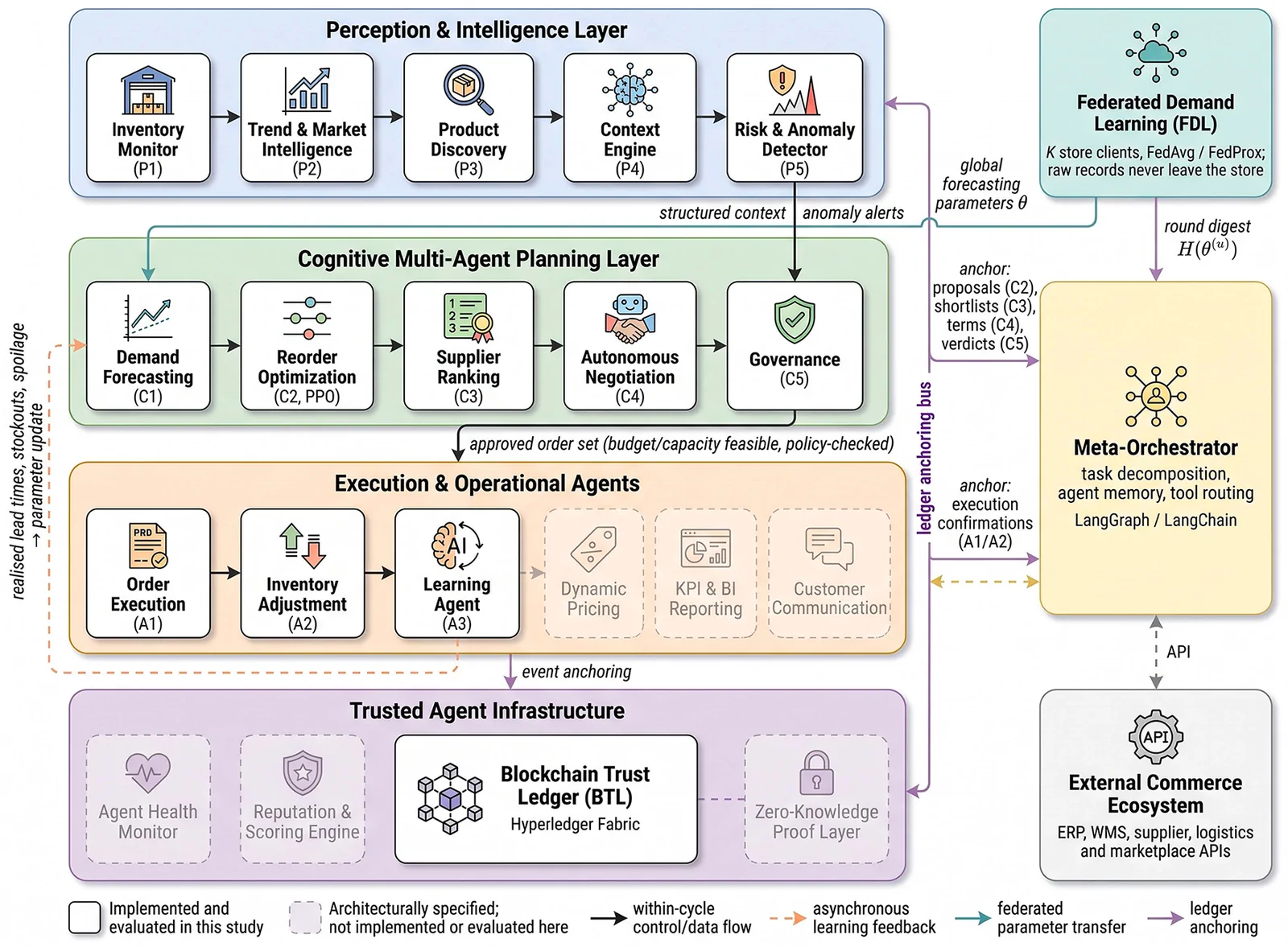
Architecture of the AAIRM framework. Solid boxes denote components implemented and evaluated in this study, whereas faded dashed boxes denote architecturally specified but unevaluated components. Black, orange, teal, and purple arrows indicate within-cycle control/data flow, asynchronous learning feedback, federated parameter transfer, and ledger anchoring, respectively. The Zero-Knowledge Proof layer is faded because it is specified architecturally but not implemented or evaluated in this study.

### 3.1 Perception layer

Five specialized agents structure the raw signals required for downstream reasoning. The Inventory Monitor Agent (P1) queries the inventory database, enterprise resource planning system, and warehouse management system for on-hand, reserved, and in-transit stock, inbound orders, and recent receipts or shrinkage, flagging at-risk SKUs against configurable thresholds. The Trend and Market Intelligence Agent (P2) ranks candidate products by momentum from search, social-media, and marketplace signals, and the Product Discovery Agent (P3) cross-references those against the existing assortment to surface underrepresented SKUs. The Context Engine (P4) combines historical sales trajectories with calendar, seasonal, price, and category metadata. The Risk and Anomaly Detector (P5) identifies demand spikes, supplier anomalies, and inventory discrepancies and then routes alerts to the Governance Agent (C5) before any procurement decision is finalized. Those alerts feed the anomaly-hold operator of Section 3.2.4.

### 3.2 Conceptualization layer

#### 3.2.1 Demand forecasting agent (C1)

C1 receives the focused SKU set from P1, trend priors from P2–P3, and contextual features from P4. For SKU *i* at time *t*, it constructs a feature vector **x**_*i, t*_ and forecasts demand *y*_*i, t*+*h*_ over horizon *H*:


$$\begin{array}{l} {ŷ}_{i,t+h}=f_{\theta }\left({\text{x}}_{i,t},h\right), \end{array}$$ (2)

where *f*_θ_ is a learned deep time-series architecture with exogenous feature attention: an LSTM encoder-decoder (60-day context, 7-day horizon) in the simulation studies, and a Temporal Fusion Transformer (90-day context, 28-day horizon) for the M5 evaluation of Section 4.14. Training minimizes the following loss function (Equation 3):


$$\begin{array}{l} L\left(\theta \right)=\underset{i,t,h}{\sum }\ell \left(y_{i,t+h},f_{\theta }\left({\text{x}}_{i,t},h\right)\right), \end{array}$$ (3)

with ℓ(·) mean squared error for point forecasts or pinball loss for quantiles. In multi-store deployments, θ comes from the Federated Demand Learning protocol of Section 3.5 rather than centralized training.

#### 3.2.2 Reorder optimization agent (C2)

For SKU *i*, let *Q*_*i*_ be the planned order quantity, ${\tilde{D}}_{i}$ the random demand during the replenishment cycle, *c*_*i*_ the unit cost, *h*_*i*_ the holding-cost rate, *p*_*i*_ the unmet-demand penalty, and δ_*i*_ the spoilage-cost rate for perishables. The single-period expected cost is


$$\begin{array}{l} C_{i}\left(Q_{i}\right)=c_{i}Q_{i}+h_{i}E\left[{\left(Q_{i}-{\tilde{D}}_{i}\right)}_{+}\right]\\ \;+p_{i}E\left[{\left({\tilde{D}}_{i}-Q_{i}\right)}_{+}\right]+\delta_{i}E\left[\max\left\{0,Q_{i}-{\tilde{D}}_{i}^{\text{life}}\right\}\right], \end{array}$$ (4)

where (*x*)_+_ = max(0, *x*) and ${\tilde{D}}_{i}^{\text{life}}$ is demand realizable within the effective shelf-life window. Perishable SKUs draw a category-specific shelf life from a truncated exponential (Supplementary Table S2). Non-perishable categories take infinite shelf life and δ_*i*_ = 0. C2 solves


$$\begin{array}{l} \underset{Q_{i}\geq 0}{\min}\underset{i}{\sum }C_{i}\left(Q_{i}\right)\;\text{subject to}\;\underset{i}{\sum }c_{i}Q_{i}\leq B,\;\underset{i}{\sum }v_{i}Q_{i}\leq V, \end{array}$$ (5)

where *B* is the purchasing budget and *V* the warehouse-capacity bound (Supplementary Table S2). In the multi-period setting, C2 learns a policy $\pi_{\phi }:S\to A$ maximizing the following expected discounted return (Equation 6):


$$\begin{array}{l} \underset{\pi_{\phi }}{\max}E\left[\overset{\infty }{\underset{t=0}{\sum }}\gamma^{t}r\left(s_{t},a_{t}\right)\right], \end{array}$$ (6)

where *r*(·, ·) is the negative expected cost of Equation 4. The per-SKU state *s*_*i, t*_ concatenates inventory position, the forecast vector and its uncertainty, remaining shelf life with recent spoilage and stockout fractions, the lead-time estimate and supplier reliability, and a shared global context *g*_*t*_ carrying budget utilization, capacity utilization, and a category indicator, so that *s*_*t*_ = ({_*s*_*i, t*_}*i*_, *g*_*t*_). Supplementary Section S5 gives the full specification. Two design points matter downstream. First, the per-SKU factored policy applies the same network to every SKU with *g*_*t*_ broadcast to each, keeping the parameter count independent of SKU count, which is the mechanism by which the policy scales from 100 to 500 SKUs. Second, the action is a squashed Gaussian mapped onto $\left[0,Q_{i}^{\max}\right]$, and it passes through the Governance Agent's projection operators (Section 3.2.4) before it is applied. The constraints of Equation 5 are therefore enforced structurally rather than learned implicitly, and C2 trains against the projected outcome. The per-epoch reward is $r\left(s_{t},a_{t}\right)=-\underset{i}{\sum }C_{i}\left(Q_{i,t}\right)$. Optimization uses PPO (Schulman et al., 2017) with generalized advantage estimation (Sutton and Barto, 2018). The actor–critic architecture and all hyperparameters are in Supplementary Table S2.

#### 3.2.3 Supplier ranking agent (C3) and autonomous negotiation agent (C4)

For each approved pair (*i, Q*_*i*_), C3 queries supplier catalogs and scores each feasible supplier $j\in S_{i}$ (unit cost *c*_*ij*_, lead time *L*_*ij*_, reliability *r*_*ij*_, minimum order quantity *m*_*ij*_) as


$$\begin{array}{l} {\text{Score}}_{ij}=\alpha_{1}c_{ij}+\alpha_{2}{\hat{L}}_{ij}-\alpha_{3}r_{ij}+\alpha_{4}I\left\{Q_{i}<m_{ij}\right\}, \end{array}$$ (7)

where ${\hat{L}}_{ij}$ incorporates historical lead-time deviations, and α_1_, …, α_4_ are tunable weights (Supplementary Table S2). Suppliers are ranked ascending by Score_*ij*_, and a shortlist passes to C4. C4 enters structured dialogue with supplier systems to obtain revised quotations, discounts, or delivery schedules, then routes confirmed proposals to C5.

#### 3.2.4 Governance agent (C5): operator-level specification

The Governance Agent underpins this study's accountability claims, so we specify it as a composition of explicit operators. Let ${\text{Q}}^{\text{neg}}={\left\{Q_{i}^{\text{neg}}\right\}}_{i\in I_{t}}$ be the negotiated quantities from C4 at cycle *t*, *c*(*i*) the category of SKU *i*, and *z*(*i*)∈{frozen, ambient} its storage zone. C5 applies four quantity-modifying operators in sequence and raises two governance flags, producing ${\text{Q}}^{\text{app}}=\left(\Pi_{\text{life}}•\Pi_{\text{cap}}•\Pi_{\text{bud}}•\Pi_{\text{hold}}\right){\text{Q}}^{\text{neg}}$. The operators fall into three functional families, and the decomposition in Section 4.6 follows exactly this partition.

*Escalation and risk holds*. The anomaly-hold operator zeroes any SKU flagged by P5 for inventory discrepancy or data-quality failure, $\Pi_{\text{hold}}:Q_{i}\mapsto Q_{i}\cdot I\left\{i\notin A_{t}\right\}$, where $A_{t}$ is the anomaly set. Separately, any surviving order whose value exceeds the human-approval threshold Θ is marked for mandatory human review, η_*i*_ = *I*{*c*_*i*_*Q*_*i*_>Θ}, with Θ = *$*50, 000 in all reported experiments.

*Feasibility projection*. Two operators enforce the hard resource ceilings. The category-budget operator truncates each order to the residual budget of its category, processing SKUs in ranked sequence. The budget-feasibility operator is defined as follows (Equation 8):


$$\begin{array}{l} \Pi_{\text{bud}}:\;Q_{i}\mapsto \min\left\{Q_{i},\frac{(B_{c\left(i\right)}-\sum_{j≺i,c\left(j\right)=c\left(i\right)}c_{j}Q_{j}{)}_{+}}{c_{i}}\right\}, \end{array}$$ (8)

where $\underset{c}{\sum }B_{c}=B$ and the default allocation is uniform across the five categories. The storage-zone operator applies the analogous truncation on volume within each temperature zone. The storage-capacity feasibility operator is defined as follows (Equation 9):


$$\begin{array}{l} \Pi_{\text{cap}}:\;Q_{i}\mapsto \min\left\{Q_{i},\frac{(V_{z\left(i\right)}-\sum_{j≺i,z\left(j\right)=z\left(i\right)}v_{j}Q_{j}{)}_{+}}{v_{i}}\right\}, \end{array}$$ (9)

with *V*_frozen_+*V*_ambient_ = *V*. Together, these two operators guarantee that the submitted order set satisfies Equation 5 on every cycle, which is the sense in which AAIRM's feasibility is hard rather than expected.

*Cross-category policy rules*. The shelf-life alignment operator caps perishable orders at the quantity that expected demand can absorb before expiry, $\Pi_{\text{life}}:Q_{i}\mapsto \min\left\{Q_{i},\;\kappa {\bar{d}}_{i}\tau_{i}\right\}$ for *i* perishable, where ${\bar{d}}_{i}$ is mean daily demand, τ_*i*_ is remaining shelf life, and κ = 0.80 is the shelf-life safety margin. The supplier-concentration rule raises a diversification flag whenever a single supplier's share of a category's approved order value exceeds ψ = 0.60. C5 also tracks the per-category ratio of stockout-penalty cost to procurement-plus-holding cost and escalates when it leaves historical bounds. Every decision (approval, modification, hold, or rejection), with its flag vector, is recorded in the Blockchain Trust Ledger (Section 3.4).

### 3.3 Action layer and learning loop

Three agents close the learning loop. The Order Execution Agent (A1) converts approved order proposals into purchase orders, arranges logistics, and creates receiving operations. The Inventory Adjustment Agent (A2) reconciles physical receiving against system records. The Learning Agent (A3) updates the forecaster of Equation 2 from realized lead times, stockouts, wastage, and demand surprises, together with the cost and reliability parameters of Equations 4, 7. Within C2's PPO loop, the temporal-difference critic loss is defined as follows (Equation 10)


$$\begin{array}{l} L_{\text{TD}}\left(\phi \right)={\left(r_{t}+\gamma V_{\phi }\left(s_{t+1}\right)-V_{\phi }\left(s_{t}\right)\right)}^{2}, \end{array}$$ (10)

the value-function component of the PPO objective (Schulman et al., 2017), tracked by A3. Three further agents, namely, Dynamic Pricing, Key Performance Indicator and Business Intelligence Reporting, and Customer Communication, are architecturally specified in the deployment template but not empirically evaluated here.

### 3.4 Blockchain trust ledger

The Blockchain Trust Ledger supplies tamper-evident decision provenance: financially meaningful pipeline events are logged and replicated in a hash-chained ledger that no single party, including the orchestrator, can edit or delete without detection. It follows the execute-order-validate architecture of Hyperledger Fabric (Androulaki et al., 2018), with four organizations representing the natural trust domains of a retail procurement network (retailer, supplier consortium, logistics provider, and independent auditor), one peer each, and Raft consensus among three ordering nodes. The permissioned design follows Saberi et al. (2019): Records are commercially sensitive, participants are identified legal entities, and throughput requirements exceed what public proof-of-work chains provide. Five event types are anchored: order proposals from C2, supplier shortlists from C3, negotiated terms from C4, governance verdicts and flag vectors from C5, and execution confirmations from A1/A2. Each event *e*_*n*_ is committed as 〈type, agent ID, *t, H*(payload), *H*(*e*_*n*−1_)〉, where *H*(·) is SHA-256. Bulky payloads are stored off-chain with only their digests anchored on-chain. An auditor verifies any historical decision by retrieving off-chain payloads, recomputing digests, and checking equality and chain continuity. A mutation of any off-chain record, or an attempted single-organization rewrite, is detected because the digests no longer match the replicated, consensus-committed chain. The Zero-Knowledge Proof layer, intended to verify commercial terms without revealing them, remains architecturally specified but unevaluated and is excluded from this study's claims.

#### 3.4.1 Adversary model and security boundary

Reporting a ledger without stating the adversary, it defends against invites overreading. The assets to be protected are the integrity and non-repudiation of the decision record, and its binding to the agent and timestamp that produced it. Confidentiality of commercial terms is out of scope since payloads are held off-chain and the Zero-Knowledge Proof layer that would address selective disclosure is unevaluated. Availability is out of scope in a specific sense. Anchoring is asynchronous, so a ledger outage delays the audit trail rather than halting procurement. That leaves the system operational but unaccountable, which is itself a governance risk.

We assume identified legal entities under a Fabric membership service provider, an honest auditor organization, and a correctly configured endorsement policy. Table 2 then states what the evaluated design withstands and what it does not. The central limitation deserves emphasis rather than a footnote. Raft (Ongaro and Ousterhout, 2014) is crash fault tolerant, not Byzantine fault tolerant. With three orderers, the deployment tolerates a single crash, so a coalition controlling the ordering service and a majority of endorsing peers can rewrite history without external detection. Defending against that adversary requires Byzantine-fault-tolerant ordering (Castro and Liskov, 1999; Barger et al., 2021), mandatory auditor endorsement of every governance verdict, and periodic notarization of block hashes outside the consortium's control. Each is a configuration change rather than a new capability, but none is evaluated here, and we claim no resistance to majority collusion.

**Table 2:** Adversary model for the Blockchain Trust Ledger.

Adversary class	Status	Outcome and residual exposure
Compromised orchestrator or single insider altering an off-chain payload	Evaluated	Detected: recomputed digest diverges from the replicated on-chain value. This is the class the 500-case replay exercises, and detection follows from SHA-256 collision resistance rather than from the experiment.
Single malicious organization rewriting its local ledger copy	Analyzed	Rejected at validation: the divergent state fails the endorsement policy and consensus check. Depends on an endorsement policy that requires signatures from more than one organization; misconfiguration removes the guarantee.
Colluding majority of organizations including the ordering service	Not mitigated	History can be rewritten without in-system detection. Requires Byzantine-fault-tolerant ordering, mandatory auditor endorsement, and external notarization of block hashes. Not evaluated.
Denial of service against the ordering service	Not mitigated	Raft tolerates one crashed orderer of three. Anchoring is asynchronous, so procurement continues while the audit trail lags, leaving decisions temporarily unaccountable. Mitigation is more orderers across administrative domains plus queue monitoring.
Off-chain payload store compromise or deletion	Partially analyzed	Detected as missing evidence rather than silent alteration, so integrity holds but evidentiary availability does not. Mitigation is per-organization replication of the payload store.
Chaincode-level exploit or logic flaw	Not assessed	Governance rules encoded in chaincode are trusted code; a flaw there compromises enforcement regardless of consensus. Requires code audit and, ideally, formal verification.
Supplier-side prompt injection into C4 (Greshake et al., 2023)	Not assessed	Malicious supplier text could steer negotiation outputs. Structured-schema validation and the human-approval threshold bound the exposure but were not tested adversarially.

“Evaluated” marks classes exercised on the four-organization testbed; “analyzed” marks classes for which the mitigation follows from the platform's documented properties but was not tested in this study. The mutation-injection replay of Section 4.12 exercises only the first row.

### 3.5 Federated demand learning

In multi-store networks, C1's parameters θ are trained via Federated Demand Learning rather than centralized pooling. Each store *k*∈{1, …, *K*} holds a private dataset $D_{k}$ and computes local empirical risk $F_{k}\left(\theta \right)=\frac{1}{\left|D_{k}\right|}\underset{\left(\text{x},y\right)\in D_{k}}{\sum }\ell \left(y,f_{\theta }\left(\text{x}\right)\right)$. The global objective is the data-weighted average. The global federated learning objective is defined as follows (Equation 11):


$$\begin{array}{l} \underset{\theta }{\min}F\left(\theta \right)=\overset{K}{\underset{k=1}{\sum }}\frac{\left|D_{k}\right|}{\sum_{k^{\prime }}|D_{k^{\prime }}|}F_{k}\left(\theta \right). \end{array}$$ (11)

Training follows federated averaging (McMahan et al., 2017): The coordinator broadcasts θ^(*u*)^, each store performs *E* local epochs to obtain $\theta_{k}^{\left(u+1\right)}$ and aggregates. The global model is updated through federated averaging as follows (Equation 12):


$$\begin{array}{l} \theta^{\left(u+1\right)}=\overset{K}{\underset{k=1}{\sum }}\frac{\left|D_{k}\right|}{\sum_{k^{\prime }}|D_{k^{\prime }}|}\theta_{k}^{\left(u+1\right)}. \end{array}$$ (12)

Because store-level category mixes and seasonality differ, client distributions are non-IID, which degrades FedAvg convergence (Kairouz et al., 2021). We therefore also evaluate FedProx (Li et al., 2020b), which adds a proximal term $\frac{\lambda }{2}∥\theta_{k}-\theta^{\left(u\right)}{∥}^{2}$ to limit client drift. Each round's aggregated digest *H*(θ^(*u*+1)^) is anchored in the BTL, so a later dispute about which model version produced a forecast is resolvable against the ledger.

#### 3.5.1 Privacy model, client heterogeneity, and what federation does not provide

Federation is often described as although withholding raw data were equivalent to privacy. It is not. What the protocol establishes is narrow and real: Point-of-sale records never leave the store, which addresses the contractual and competitive objections that block data pooling among franchisees. What it does not establish is confidentiality of the information carried in the updates. Under an honest-but-curious coordinator, each $\theta_{k}^{\left(u+1\right)}$ is observed individually, and gradient-inversion attacks have reconstructed training samples from exactly this signal (Zhu et al., 2019; Geiping et al., 2020), while membership inference can reveal whether a record contributed to training (Shokri et al., 2017). For a retail forecaster, the exposure is commercial rather than personal, since the recoverable signal concerns store-level demand and promotional response, but a competitor-adjacent coordinator is precisely the threat that motivated federation in the first place.

Three mechanisms close that gap, and a deployment should be explicit about which one it adopts. Secure aggregation (Bonawitz et al., 2017) reveals only the sum, which neutralizes per-client inversion at modest communication cost. Differentially private training (Abadi et al., 2016; McMahan et al., 2018) bounds what any observer can infer, at an accuracy cost that has to be measured rather than assumed, particularly for the sparse intermittent SKUs where federation delivers most of its benefit. Robust aggregation such as coordinate-wise median or Krum (Blanchard et al., 2017) addresses a different adversary, a client poisoning the global model (Bagdasaryan et al., 2020). We implement and evaluate none of the three, so we make no formal privacy claim. The federated results below quantify the accuracy and downstream cost of decentralization, not the strength of a guarantee.

A Dirichlet partition of category mix with concentration β = 0.5 across *K* = 8 stores induces client heterogeneity. Each store ends up with a moderately skewed assortment: It covers all five categories, but the proportions differ substantially. This matches multi-banner general-merchandise retail. It does not match the harder regime of single-category specialists or strongly regional assortments, which a concentration below β = 0.1 would represent and which we do not evaluate. Because the FedAvg-to-FedProx convergence gap is itself a function of heterogeneity, our reported 0.5-point separation should be read as a moderate-heterogeneity result, and we expect sharper partitions to widen it.

### 3.6 Use-case: execution flow

Supplementary Figure S4 traces a representative replenishment request end to end. The Meta-Orchestrator routes the request to P1, which confirms a low-stock condition. C1 and C2 compute the forecast and the proposed quantity, and C3 and C4 return ranked and then negotiated supplier terms. C5 issues a feasibility and policy verdict and anchors it in the BTL alongside its approve, modify, or reject decision. A1 and A2 then execute, and the execution is anchored in turn.

## 4 Experimental setup and results

### 4.1 Experimental environment and configuration

Each logical agent was instantiated as a discrete LangChain agent with a defined tool set: an inventory and sales database connector, a supplier catalog query service, a logistics interface stub, and a warehouse-management adapter. Numerical forecasting and optimization routines were implemented in Python and invoked as tools. Agent reasoning was supported by GPT-4o-mini (OpenAI, gpt-4o-mini-2024-07-18) under the settings in Supplementary Table S2. GPT-4o-mini exposes no user-settable seed, so exact token-level reproduction across calls is not guaranteed. LLM-backed agents operate primarily as structured orchestrators over predefined tool calls, and whether semantic reasoning adds measurable value is assessed directly in the ablation of Section 4.5.

Demand was simulated with category-specific base profiles, weekly seasonality, promotional uplifts, and independent random shocks. Perishable SKUs drew shelf lives from a truncated exponential distribution and lead times from per-category truncated normal distributions, with three to five suppliers per SKU. The primary 100-SKU experiment spanned grocery (30 SKUs), frozen food (20), apparel (15), cosmetics (20), and dry fruits (15). The 500-SKU experiment used the same five categories at 100 SKUs each. All distributional parameters are in Supplementary Table S2. Experiments ran on an AMD EPYC 7763 (64 cores), 256 GB RAM, and an NVIDIA A100 (40 GB), taking approximately 4.2 h per seed at 100 SKUs and 22.8 h per seed at 500 SKUs. The simulation code and the full statistical specification are available in the public repository (https://github.com/aliakarma/AAIRM).

The BTL evaluation deployed the four-organization permissioned network (one peer each and three Raft orderers) on the same workstation using containerized peers (0.5 s batch timeout and 50-transaction batch size), replaying anchoring events recorded from the 100-SKU simulation at controlled rates. The FDL evaluation partitioned the 100-SKU demand environment across *K* = 8 simulated stores with non-IID category mixes (Dirichlet concentration β = 0.5, see Section 3.5.1). Each store trained a local LSTM encoder-decoder, and FedAvg and FedProx (λ = 0.01) ran for 60 rounds with *E* = 2 local epochs. Comparators were centralized training and local-only training. Accuracy is weighted absolute percentage error (WAPE) on a held-out horizon, and downstream impact is measured by re-running the full AAIRM pipeline with each variant's forecaster.

### 4.2 Baselines

AAIRM was compared against seven baselines spanning classical practice, machine-learning-augmented rules, reinforcement learning, and agentic-LLM design. **BL1 (ROP–EOQ)** triggers orders below a computed reorder point using safety factor *z* = 1.645, a 95% target cycle-service level, with μ_*D*_ and σ_*D*_ estimated from a 30-period rolling window. **BL2 (ML + Static)** supplies XGBoost demand estimates to unchanged, non-adaptive order-quantity rules with no supplier negotiation or cross-category coordination. It represents a documented industry anti-pattern rather than the strongest non-agentic comparator. That role belongs to **BL3 (ML + Adaptive)**, which strengthens BL2 by recalculating safety stock each period as $z\cdot {\hat{\sigma }}_{i,t}$ from rolling forecast-residual variance. **BL4 (Per-SKU DQN)** follows Oroojlooyjadid et al. (2022): independent DQN agents per SKU with no cross-SKU coordination, governance, or negotiation. **BL5 (MAPPO)** is a shared-parameter multi-agent PPO policy with a centralized critic (Yu et al., 2022), applied to the same decision process as C2 but without governance (C5), supplier ranking (C3), or ledger anchoring. **BL6 (MAPPO + projection)** is BL5 with C5's feasibility operators applied post-hoc. BL5 has no feasibility mechanism of its own, so an AAIRM-vs.-BL5 comparison would confound learned coordination with projection. BL6 exists solely to separate the two. **BL7 (LLM-Agent)** is an InvAgent-style (Quan and Liu, 2024) architecture in which LLM-driven agents negotiate quantities and supplier terms through natural-language reasoning rather than a learned policy or projection step, giving the LLM-orchestration findings an external comparison against a published agentic design.

### 4.3 Evaluation protocol, comparability, and statistical reporting

Each seed instantiates an independent 730-day demand trajectory. The first 365 days fit the forecaster and train the policy. The final 365 days form the held-out evaluation horizon on which every reported metric is computed. One RL episode is one pass through a 365-day trajectory, so the 200 training episodes used at both scales correspond to approximately 7.3 × 10^4^ environment steps per seed. Each seed therefore contributes exactly one evaluation replication over a common horizon, and reported means and standard deviations are taken across seeds: ten (42–51) at 100 SKUs and five (42–46) at 500 SKUs and on M5. All policies within a scale are evaluated on the identical set of seed-matched demand trajectories, which is what makes the paired tests below valid.

Significance testing uses paired *t*-tests on seed-level normalized cost differences at α = 0.05, with Holm–Bonferroni correction within each family of pairwise comparisons. Shapiro–Wilk tests confirmed normality of the seed-level differences for all primary comparisons (*W*≥0.91, *p*≥0.28). Confidence intervals come from the corresponding paired standard errors. Because BL1 is identically 1.000 per seed by construction, the AAIRM-vs.-BL1 difference has the same dispersion as AAIRM's own normalized cost, and its effect size $d=t/\sqrt{n}=9.42$ (Cohen, 1988) is a mechanical consequence of that zero-variance reference rather than a conventional two-sample effect size. We flag this once and do not restate it. For the LLM orchestrator's null result, we additionally run a two one-sided tests (TOST) equivalence procedure against a pre-specified ±2 percentage-point margin, the smallest cost difference judged to matter for a deployment decision.

Comparability across policies deserves an explicit statement, since a cost comparison between an unconstrained and a constrained policy is meaningless if they are granted different resources. Every one of the eight policies faces the identical per-period purchasing budget *B* and warehouse-capacity ceiling *V* (Supplementary Table S2), and any order set jointly exceeding either ceiling is proportionally scaled down before execution regardless of which policy produced it. That shared scaling is a safety net applied uniformly, not a feature of AAIRM. Supplementary Table S6 records, policy by policy, the two places they legitimately differ: the knob available for setting service level, and whether any feasibility mechanism acts before submission. The infeasible-submission statistic reported in Section 4.7 is measured on the order set each policy submits, immediately before that shared scaling. That is the comparison of interest: whether a policy ever asks for more than the envelope allows.

Two consequences should be stated rather than left for a reader to infer. First, service-level parity in the 95–98% band is obtained by moving AAIRM along its own cost–service frontier to meet the baselines, not by retuning the baselines to meet AAIRM. This is the conservative direction for our claim, because each baseline is compared at the operating point its own design produces, while AAIRM has to buy additional service at increasing marginal cost. Second, and against our own interest, BL1 and BL3 were held at the conventional *z* = 1.645 rather than at the cost-minimizing *z* for this environment. Total cost under ROP–EOQ is not monotone in *z*, so a cost-optimal retuning would narrow the 13.2-point gap measured at AAIRM's leanest operating point, although it would also push BL1 below the 95% service level its design targets. The comparisons we regard as decisive are therefore the matched-service ones of Section 4.10.

### 4.4 Primary evaluation: 100 SKUs, 10 seeds

Table 3 presents overall performance at 100 SKUs. AAIRM achieves the lowest mean total cost [0.868 ± 0.014, 95% CI (0.858, 0.878)], a 13.2% reduction against BL1 and 23.3% against BL2, with *t*_(9)_ = 29.8 and *t*_(9)_ = 6.83, respectively (*p* < 0.001, both surviving Holm–Bonferroni correction, and the BL1 test is equivalently a one-sample test against 1.000). BL3 reaches 0.962 ± 0.031, a 3.8% improvement over BL1 that still leaves a 9.4-point gap to AAIRM [*t*_(9)_ = 9.12, *p* < 0.001]. Adaptive safety stock therefore corrects most of BL2's overstocking pathology, and the residual gap reflects the added value of RL ordering and coordinated governance. BL2's 13.2% cost increase over BL1 comes from overstocking slow-moving categories because its static rule never updates reorder thresholds from forecast residuals. In apparel, it carries 25.60 units per SKU against BL1's 11.15. AAIRM reduces average inventory by 28.6% and spoilage by 22.0% relative to BL1, with cross-seed dispersion more than eight times tighter than BL2's.

**Table 3:** Overall performance across inventory policies, 100-SKU primary evaluation, and mean ± SD over 10 seed-matched trajectories (seeds 42–51).

Policy	Stockout (%)	Fill rate (%)	Avg. inv.	Total cost [95% CI]	Spoilage (%)
BL1 (ROP–EOQ)	1.19 ± 0.31	98.81 ± 0.31	7.10 ± 0.26	1.000 ± 0.000	5.85 ± 0.54
BL2 (ML + Static)	4.86 ± 3.77	95.14 ± 3.77	7.41 ± 1.77	1.132 ± 0.118 [1.048, 1.216]	5.58 ± 1.44
BL3 (ML + Adaptive)	2.84 ± 0.62	97.16 ± 0.62	6.43 ± 0.29	0.962 ± 0.031 [0.940, 0.984]	5.41 ± 0.58
AAIRM (proposed)	7.71 ± 0.78	92.29 ± 0.78	**5.07 ± 0.16**	**0.868 ± 0.014** [0.858, 0.878]	**4.56 ± 0.41**

Total cost is normalized to BL1 per seed, which therefore has zero dispersion by construction. Bracketed values are 95% confidence intervals on the mean. Bold values indicate the best-performing result for each metric. For stockout, total cost, and spoilage, lower values are preferable; for fill rate, higher values are preferable. Average inventory is reported in units per SKU.

AAIRM's default operating point (92.29% fill) sits below the 95%–98% range typical of retail service-level agreements, and we treat it as the leanest-cost point rather than the deployable one. BL1's low stockout rate comes from carrying substantially more inventory, the canonical overstocking behavior of conservative ROP–EOQ policies. Its realized 98.81% fill exceeds its nominal 95% target because the 30-period rolling safety-stock estimate of Equation 1 lags demand shifts under comparatively smooth demand and overshoots the target quantile. Performance is consistent across all 10 seeds, with cost from 0.852 to 0.899 and stockout from 6.34% to 8.73%. The RL training curve is shown in Supplementary Figure S1.

Table 4 collects the headline effects with interval estimates since point estimates alone understate how differently the comparisons are resolved. The comparisons against classical and machine-learning baselines are decisive, with intervals far from zero. The comparison against multi-agent RL is not: The advantage over BL5 is nominally significant but its interval nearly touches zero, and once BL5 is granted the same projection step the interval covers zero outright. The LLM orchestrator's interval spans zero and is contained within the pre-specified equivalence margin at the 90% level, which is the basis for the equivalence claim rather than a bare failure to reject.

**Table 4:** Headline effects with 95% confidence intervals.

Comparison	Scale	*n*	Δ (cost points)	95% CI	Relative (%)
AAIRM vs. BL1 (ROP–EOQ)	100 SKU	10	0.132	[0.122, 0.142]	13.2 [12.2, 14.2]
AAIRM vs. BL2 (ML + static)	100 SKU	10	0.264	[0.177, 0.351]	23.3 [15.6, 31.0]
AAIRM vs. BL3 (ML + adaptive)	100 SKU	10	0.094	[0.071, 0.117]	9.8 [7.3, 12.2]
AAIRM vs. BL5 (MAPPO)	100 SKU	10	0.017	[0.001, 0.033]	1.9 [0.1, 3.7]
AAIRM vs. BL6 (MAPPO + projection)	100 SKU	10	0.011	[−0.004, 0.026]	1.2 [−0.4, 2.9]
LLM orchestrator (D vs. C)	100 SKU	10	0.006	[−0.009, 0.021]	0.6 [−0.9, 2.1]
AAIRM vs. BL1, excl. dry fruits	500 SKU	5	0.129	[0.105, 0.153]	12.9 [10.5, 15.3]
AAIRM vs. BL1, all categories	500 SKU	5	0.171	[0.150, 0.192]	17.1 [15.0, 19.2]
AAIRM vs. BL1 (M5)	M5	5	–	–	10.2 [8.0, 12.4]

All intervals are computed from the seed-level paired differences underlying the significance tests reported elsewhere in this section; no additional experiments were run to produce them. Δ is expressed in normalized cost points relative to BL1, where 0.132 corresponds to a 13.2-point reduction. Relative figures express Δ as a percentage of the comparator's own cost. For the LLM orchestrator row, the TOST-relevant 90% interval is [−0.6, 1.8] points, entirely inside the pre-specified ±2-point margin.

### 4.5 Ablation study

To isolate AAIRM's principal components, we run the four-variant ablation of Table 5 at 100 SKUs over 10 seeds, each variant removing or replacing components while holding the rest fixed. Variant A (RL-only) replaces the LLM Meta-Orchestrator with a deterministic router, disables the Governance Agent, bypasses negotiation, and selects suppliers by lowest cost. Variant B (Governance-only) keeps the full coordination and governance stack but replaces the RL policy with ROP–EOQ. Variant C (No-LLM) keeps RL ordering, governance, supplier ranking, and negotiation, replacing only the orchestrator with a deterministic router running the same tool-call sequence. Variant D is the full system.

**Table 5:** Ablation study, 100-SKU scale, 10 seeds.

Variant	Total cost	Stockout (%)	Avg. inv. (units)	*p* vs. D
A (RL-only)	0.912 ± 0.019	6.93 ± 0.91	5.42 ± 0.19	< 0.001
B (governance-only)	0.978 ± 0.022	2.15 ± 0.48	6.71 ± 0.24	< 0.001
C (No-LLM)	0.874 ± 0.016	7.54 ± 0.82	5.12 ± 0.17	0.38
D (full AAIRM)	0.868 ± 0.014	7.71 ± 0.78	5.07 ± 0.16	–

Variant D repeats the full-system result from Table 3. The *p*-value column reports Holm-corrected paired *t*-tests of each variant against full AAIRM. Note that Variants A and C differ in three components, not one; Section 4.6 separates them.

Variant A reaches 0.912 ± 0.019, an 8.8-point reduction over BL1 and the largest single contribution, locating most of the economic value in the learned ordering policy. Variants C and D differ in exactly one component, so their comparison isolates the LLM orchestrator cleanly. The contribution is 0.6 points, not statistically significant (*p* = 0.38), and confirmed practically equivalent by TOST at a ±2-point margin. We read this as a boundary condition rather than a general verdict on language-model orchestration. The simulated environment exposes fully structured state and a deterministic tool interface, which is the regime in which a rule-based router should match an LLM. Settings with unstructured supplier communication or ambiguous exceptions lie outside this evaluation. Variant B, at 0.978 ± 0.022, shows modest but real savings from coordinated governance even without a learned policy.

Variants A and C differ in three respects, not one: A has no governance, no negotiation, and no supplier ranking, while C has all three. Their 3.8-point difference is therefore the contribution of the entire coordination-and-governance block, and attributing it to governance alone conflates three mechanisms. Section 4.6 separates them. The additive reading (8.8+3.8+0.6 ≈ 13.2 points) is arithmetic consistency rather than a formal test of path independence, since the design omits the RL-plus-LLM-without-governance cell and interaction effects cannot be excluded.

### 4.6 Decomposing the coordination and governance block

The operator specification of Section 3.2.4 partitions C5 into three functional families, with supplier ranking (C3) and negotiation (C4) separable alongside them. That structure supports a layered ablation adding one mechanism at a time from Variant A to Variant C, so every increment is attributable to a named operator rather than a block (Table 6).

**Table 6:** Layered decomposition of the coordination-and-governance block at 100-SKU scale, 10 seeds.

Configuration	Mechanism added	Total cost	Δ (pts)	Infeas. subm. (%)	Stockout (%)
G0 (=variant A)	None (RL ordering only)	0.912 ± 0.019	–	8.6 ± 1.7	6.93 ± 0.91
G1	Feasibility projection Π_bud_, Π_cap_	0.898 ± 0.018	1.4	**0.0 ± 0.0**	7.21 ± 0.88
G2	Anomaly hold Π_hold_ and escalation Θ	0.895 ± 0.018	0.3	0.0 ± 0.0	7.28 ± 0.85
G3	Cross-category rules Π_life_, ψ	0.884 ± 0.017	1.1	0.0 ± 0.0	7.46 ± 0.84
G4	Supplier ranking (C3)	0.878 ± 0.016	0.6	0.0 ± 0.0	7.51 ± 0.83
G5 (=variant C)	Autonomous negotiation (C4)	0.874 ± 0.016	0.4	0.0 ± 0.0	7.54 ± 0.82

Each row adds one mechanism to the row above. G0 is Variant A and G5 is Variant C from Table 5, so the increments sum to the 3.8-point block difference. “Infeasible submissions” is the percentage of decision cycles in which the order set submitted for execution would have breached *B* or *V* absent the shared proportional-scaling safety net. Bold entries indicate the results corresponding to the proposed work (AAIRM framework).

Three readings follow. Feasibility projection is the largest governance mechanism at 1.4 points, and the only one that changes the feasibility status of the system rather than its cost alone. It takes the infeasible-submission rate from 8.6% to zero, on every cycle rather than in expectation. Cross-category policy rules contribute 1.1 points, almost all from shelf-life alignment, which caps perishable orders at what expected demand can absorb before expiry and so converts spoilage cost into a small increase in stockout. The escalation family contributes least, at 0.3 points, and the reason is worth stating precisely. In simulation, the human-approval threshold raises a flag but does not block execution, so almost all of that 0.3 points comes from anomaly holds rather than from escalation itself. Escalation's value in deployment is governance, not cost, which this experiment cannot measure.

Separating governance proper from supplier coordination gives 2.8 points for the C5 operator chain and 1.0 for C3 and C4 combined, revising our previously reported attribution of the full 3.8 points to governance. Ledger anchoring appears nowhere here because it contributes nothing to cost by construction: The BTL records decisions and never alters them, so its economic footprint is the 6.6% overhead of Section 4.12 and its benefit is auditability. Isolating that benefit numerically would require a scenario in which auditability itself has measurable value, such as dispute resolution or regulatory inspection, which lies outside this study.

### 4.7 Reinforcement-learning and agentic baseline comparison

The ablation isolates components internally. Here, we test the framework externally against strong RL and agentic-LLM comparators over 10 seeds at 100 SKUs, as summarized in Table 7.

**Table 7:** Reinforcement-learning and agentic-LLM baseline comparison, 100-SKU scale, 10 seeds.

Policy	Total cost	Stockout (%)	Fill rate (%)	Infeasible subm. (%)
BL4 (Per-SKU DQN)	0.940 ± 0.027	9.6 ± 1.9	90.4 ± 1.9	14.1 ± 2.1
BL5 (MAPPO)	0.885 ± 0.029	8.9 ± 1.3	91.1 ± 1.3	7.9 ± 1.6
BL6 (MAPPO + projection)	0.879 ± 0.026	8.4 ± 1.2	91.6 ± 1.2	**0.0 ± 0.0**
BL7 (LLM-Agent, InvAgent-style)	0.927 ± 0.028	9.4 ± 2.0	90.6 ± 2.0	15.8 ± 2.6
AAIRM (RL + governance)	**0.868 ± 0.014**	7.71 ± 0.78	**92.29 ± 0.78**	**0.0 ± 0.0**

Total cost is normalized to BL1 per seed. “Infeasible submissions” is the percentage of decision cycles in which the order set submitted for execution would have breached the shared budget or capacity ceiling absent the proportional-scaling safety net, measured after any policy-internal feasibility mechanism and before that shared net, as defined in Section 4.3. AAIRM's rate before its own projection step is 8.6% (Table 6). Bold entries indicate the results corresponding to the proposed work (AAIRM framework).

BL4, lacking cross-SKU coordination, trails both multi-agent policies and frequently proposes joint orders outside the shared envelope. BL5 closes most of the aggregate-cost gap to AAIRM but submits infeasible order sets on 7.9% of cycles. That rate is eliminated by the projection step itself rather than by AAIRM's coordination, since BL6 also reaches zero, leaving a residual 1.1-point difference. Paired tests over the 10 shared seeds give a mean AAIRM advantage over BL5 of 0.017 [1.9%, *t*_(9)_ = 2.44, nominal *p* = 0.037] and over BL6 of 0.011 [1.2%, *t*_(9)_ = 1.66, nominal *p* = 0.13]. Holm–Bonferroni correction raises the BL5 comparison to *p* = 0.074 and leaves BL6 at 0.13, so neither survives at α = 0.05, as the intervals in Table 4 show directly. We therefore claim no robust cost advantage over strong multi-agent RL. The defensible claims are the hard feasibility guarantee and the auditable decision record, both holding by construction rather than by a contested margin.

#### 4.7.1 Why projection rather than a lagrangian formulation

We did not evaluate constrained-RL comparators such as PPO-Lagrangian or MAPPO-Lagrange, nor an action-space embedding of the budget constraint. The omission is a genuine gap, but two arguments for projection are design claims rather than limitations and belong here.

The first is about what kind of guarantee each approach delivers. Lagrangian methods bound expected constraint violation over the training distribution (Altman, 1999; Achiam et al., 2017). They do not prevent violation on any particular cycle. A retailer's per-period purchasing budget and warehouse volume are hard ceilings that cannot be exceeded once on a Tuesday and compensated for on a Wednesday, so an expected-violation guarantee is the wrong instrument for this constraint class and structural enforcement (Dalal et al., 2018) is the appropriate one. The second is a governance argument we regard as at least as important. A projection operator is a fixed, human-readable rule that can be inspected, versioned, encoded at the chaincode layer, and audited against the ledger. A learned multiplier is an opaque parameter whose current value determines how strictly a commercial constraint is being enforced. For a system whose purpose is accountable autonomy, that difference is not incidental.

On cost we expect the trade-off to run modestly the other way, since a Lagrangian policy anticipates the constraint through a shaped objective rather than being truncated at execution. AAIRM captures part of that benefit already because C2 trains against the projected outcome rather than the raw proposal, which is also why in-loop projection (AAIRM) outperforms post-hoc projection (BL6) rather than merely matching it. Supplementary Section S4 sets out the remaining trade-offs, namely the multiplier tuning burden (Stooke et al., 2020) and why an action-space embedding would break the per-SKU factorization on which the 100-to-500-SKU transfer depends. A PPO-Lagrangian and MAPPO-Lagrange comparator suite remains a priority for future work, and we expect it to sharpen rather than overturn this reading.

### 4.8 Per-category analysis

Supplementary Table S3 gives the full per-category breakdown for all four fully evaluated policies. Three patterns matter. Apparel is the principal source of BL2's cost failure, stockpiling 25.60 units per SKU against BL1's 11.15. BL3 largely corrects that at 8.80, and AAIRM reaches 8.44 through leaner learned ordering. In the perishable categories, AAIRM cuts inventory by 30.0% (frozen food) and 30.9% (cosmetics) against BL1, with spoilage down 25.3% and 26.0%, respectively. Dry fruits is the most challenging category, carrying 15.20% baseline spoilage under BL1. AAIRM's 7.75% stockout and 12.03% spoilage reflect a genuine cost–service trade-off. Section 4.10 shows that the trade-off is tunable through the category-specific stockout-penalty weight, and Section 4.9 shows it can become a governance failure at larger scale if left uncorrected.

### 4.9 Scalability analysis: 500 SKUs, five seeds

We expand the simulation to 500 SKUs across five categories of 100 each over five seeds (42–46). Five seeds rather than 10 reflects the higher per-run cost at this scale, so we treat the variance estimates as indicative. AAIRM reaches a mean total cost of 0.829 ± 0.017, a 17.1% aggregate reduction over BL1 (Table 8). Excluding dry fruits, where a reward-hacking failure yields a 31.54% stockout rate, the reduction is 12.9% (95% CI 10.5–15.3), which closely matches the 13.2% at 100-SKU scale. We present 12.9% as the representative figure since the 17.1% aggregate partly reflects service-level abandonment in one category rather than genuine optimization.

**Table 8:** Overall performance at 500-SKU scale, mean ± SD over five seed-matched trajectories (seeds 42–46).

Policy	Stockout (%)	Fill rate (%)	Avg. inv. (units)	Total cost	Spoilage (%)
BL1 (ROP–EOQ)	1.23 ± 0.20	98.77 ± 0.20	7.12 ± 0.11	1.000 ± 0.000	5.99 ± 0.30
BL2 (ML + Static)	5.24 ± 0.81	94.76 ± 0.81	7.99 ± 1.23	1.203 ± 0.094	5.88 ± 0.89
BL3 (ML + Adaptive)	3.10 ± 0.71	96.90 ± 0.71	6.51 ± 0.33	0.948 ± 0.027	5.52 ± 0.61
AAIRM (all categories)	14.34 ± 1.85	85.66 ± 1.85	**4.64 ± 0.06**	**0.829 ± 0.017**	**3.51 ± 0.30**
AAIRM (excl. dry fruits)	9.90 ± 1.12	90.10 ± 1.12	4.71 ± 0.07	0.871 ± 0.019	2.96 ± 0.24

Total cost is normalized to BL1 per seed. AAIRM's all-categories spoilage and cost figures are procurement-volume-weighted averages across the five categories, not simple means of per-category percentages; dry fruits carries a 31.54% stockout rate from reward miscalibration and, despite equal SKU count, contributes a larger share of total procured units because of its faster turnover, which raises its weight in the aggregate relative to a naive per-category mean. Bold entries indicate the results corresponding to the proposed work (AAIRM framework).

Dry fruits accounts for most of the aggregate 14.34% stockout rate, through a reward miscalibration that surfaces only at scale. The spoilage penalty at weight 1.0 rewards lean ordering that eliminates spoilage at the expense of fill rate. This is the reward-misspecification failure mode documented across the alignment literature (Amodei et al., 2016; Skalse et al., 2022; Pan et al., 2022), in which an agent optimizes the stated objective while violating unstated commercial intent. The correction needs no architectural change. Raising the dry-fruits stockout-penalty weight from *w*_*p*_ = 1.2 to $w_{p}^{\text{DF}}=9.0$ lifts fill from 68.5% to 95.3%, and normalized category cost rises to 0.973, still below ROP–EOQ. The price is higher spoilage, the intrinsic tension in a category with 15.2% baseline spoilage (Table 9). All other categories hold stockout levels consistent with the primary evaluation.

**Table 9:** Dry-fruits category recalibration at 500-SKU scale via the category-specific stockout-penalty weight $w_{p}^{\text{DF}}$, five seeds per row.

$w_{p}^{\text{DF}}$	Stockout (%)	Fill rate (%)	Category cost	Spoilage (%)
1.2 (default)	31.54 ± 3.10	68.46 ± 3.10	0.742 ± 0.041	4.10 ± 0.62
3.0	14.8 ± 2.4	85.2 ± 2.4	0.859 ± 0.034	7.9 ± 0.9
6.0	7.2 ± 1.6	92.8 ± 1.6	0.927 ± 0.030	11.9 ± 1.1
9.0	4.7 ± 1.2	95.3 ± 1.2	0.973 ± 0.028	13.6 ± 1.8

Costs are normalized to BL1 on the dry-fruits category alone. The default row reproduces the 31.54% failure; recalibration trades spoilage for service monotonically.

### 4.10 Pareto-Frontier analysis of the cost–service trade-off

A recurring practitioner concern is whether AAIRM's cost advantage survives at commercially standard fill rates. We sweep the stockout-penalty weight *w*_*p*_∈{1.2, 2.0, 3.0, 5.0, 8.0} at 100 SKUs over 10 seeds, retraining C2 at each setting, and trace the frontier in Figure 2. Cost rises monotonically with service, from 7.71% stockout at 0.868 cost (*w*_*p*_ = 1.2) to 1.8% at 0.978 (*w*_*p*_ = 8.0). A commercially standard 97% fill falls between the *w*_*p*_ = 3.0 and *w*_*p*_ = 5.0 points rather than on a retrained one. At the two measured points bracketing it, AAIRM retains an 8.2-point advantage at 96.1% fill and 4.9 points at 97.4%. Linear interpolation to exactly 3% stockout gives roughly 5.9 points, which should be read as an interpolation rather than an independent measurement. Even at near-parity service (1.8% stockout against BL1's 1.19%) a small positive margin survives, so the result is not purely an artifact of service-level sacrifice. Per-point dispersion runs from ±0.014 to ±0.023, so adjacent frontier points remain distinguishable at α = 0.05.

**Figure 2:**
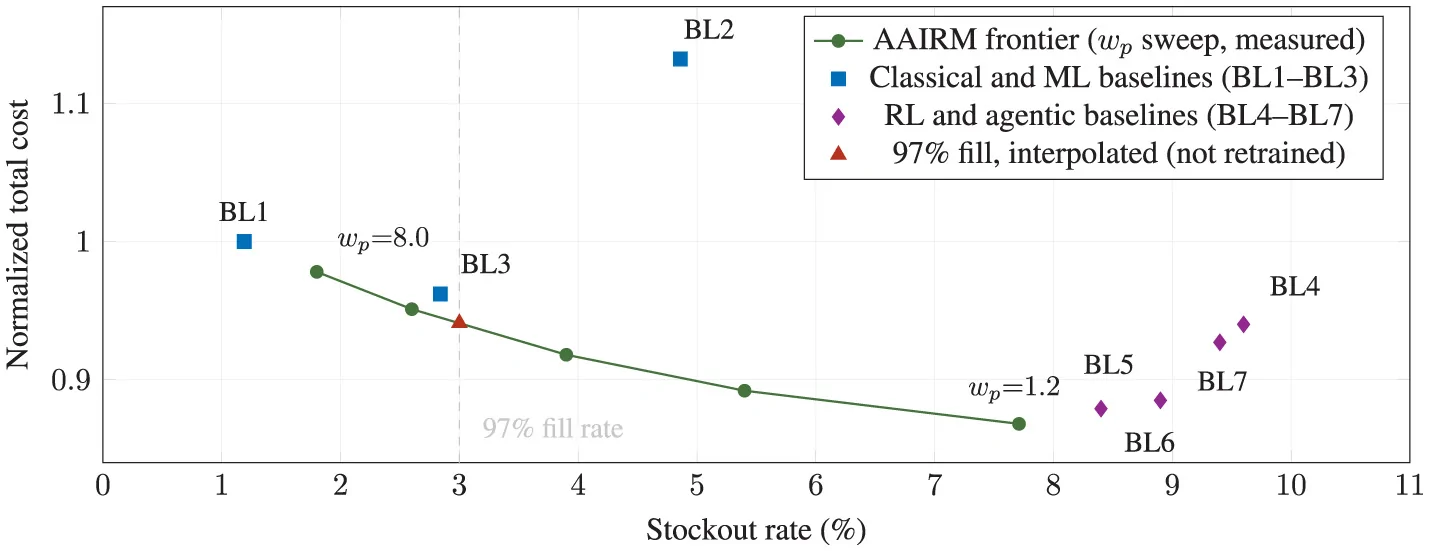
Cost–service frontier at 100-SKU scale. The AAIRM frontier is obtained by sweeping the stockout-penalty weight *w*_*p*_; baseline methods are shown at their respective operating points. Lower and further left indicates better performance. The 97% fill-rate point is linearly interpolated between measured AAIRM points.

### 4.11 Federated demand learning evaluation

Table 10 reports the eight-store non-IID testbed. Centralized training, the privacy-violating upper bound, yields a held-out WAPE of 18.4%. Local-only degrades to 24.7%, a substantial loss for sparse, seasonal SKUs. FedAvg recovers most of that gap at 19.8% and FedProx reaches 19.3% under non-IID distributions, consistent with its design objective (Li et al., 2020b), converging more smoothly than FedAvg (Supplementary Figure S2).

**Table 10:** Federated demand learning evaluation at 100-SKU scale across *K* = 8 non-IID stores, 10 seeds.

Training regime	WAPE (%)	Downstream cost [95% CI]	Raw data leaves store?
Centralized (pooled)	18.4 ± 0.6	0.868 ± 0.014 [0.858, 0.878]	Yes
FedProx (λ = 0.01)	19.3 ± 0.7	0.881 ± 0.016 [0.870, 0.892]	No
FedAvg	19.8 ± 0.8	0.886 ± 0.018 [0.873, 0.899]	No
Local-only	24.7 ± 1.4	0.917 ± 0.023 [0.901, 0.933]	No

WAPE is held-out forecast error; downstream cost is the normalized total cost of the full AAIRM pipeline using each forecaster. The final column records only whether raw records leave the store, which is a weaker property than a formal privacy guarantee.

The decisive question is downstream cost, not forecast accuracy alone. Re-running the pipeline with each forecaster gives normalized costs of 0.868 ± 0.014 (centralized), 0.881 ± 0.016 (FedProx), 0.886 ± 0.018 (FedAvg), and 0.917 ± 0.023 (local-only). The federated penalty of 1.3 to 1.8 points still leaves an 11–12% advantage over ROP–EOQ, so data-sovereign deployment is achievable at modest economic cost. Forgoing collaboration entirely retains only 8.3 of the 13.2 points. Communication cost is modest: A 1.9M-parameter forecaster transfers approximately 7.6 MB per store per round, with 60 rounds completing in under 25 min. These figures characterize the cost of decentralization, not the strength of a privacy guarantee. As Section 3.5.1 sets out, neither secure aggregation nor differentially private training was applied, so the penalty is a lower bound on what a formally private deployment would pay.

### 4.12 Blockchain trust ledger evaluation

The BTL evaluation addresses added decision latency, sustained throughput, storage consumption, and the correctness of tamper detection. Figure 3 shows the transaction-commit sequence and Table 11 the measurements. Because anchoring is asynchronous, end-to-end decision-cycle latency rises from the measured 3.2 s baseline to 3.41 s, a 6.6% overhead. Synchronous anchoring, which a deployment might require for governance verdicts specifically, would instead add the full commit latency to the critical path. The network sustains roughly 310 transactions per second before queueing delay grows super-linearly (Supplementary Figure S3), whereas a deployment making 1,000 replenishment decisions per day produces approximately 5,000 anchoring events, more than three orders of magnitude below capacity. Digest-only anchoring consumes approximately 1.9 KB of ledger state per event, projecting to roughly 3.5 GB per peer per year at that rate.

**Figure 3:**
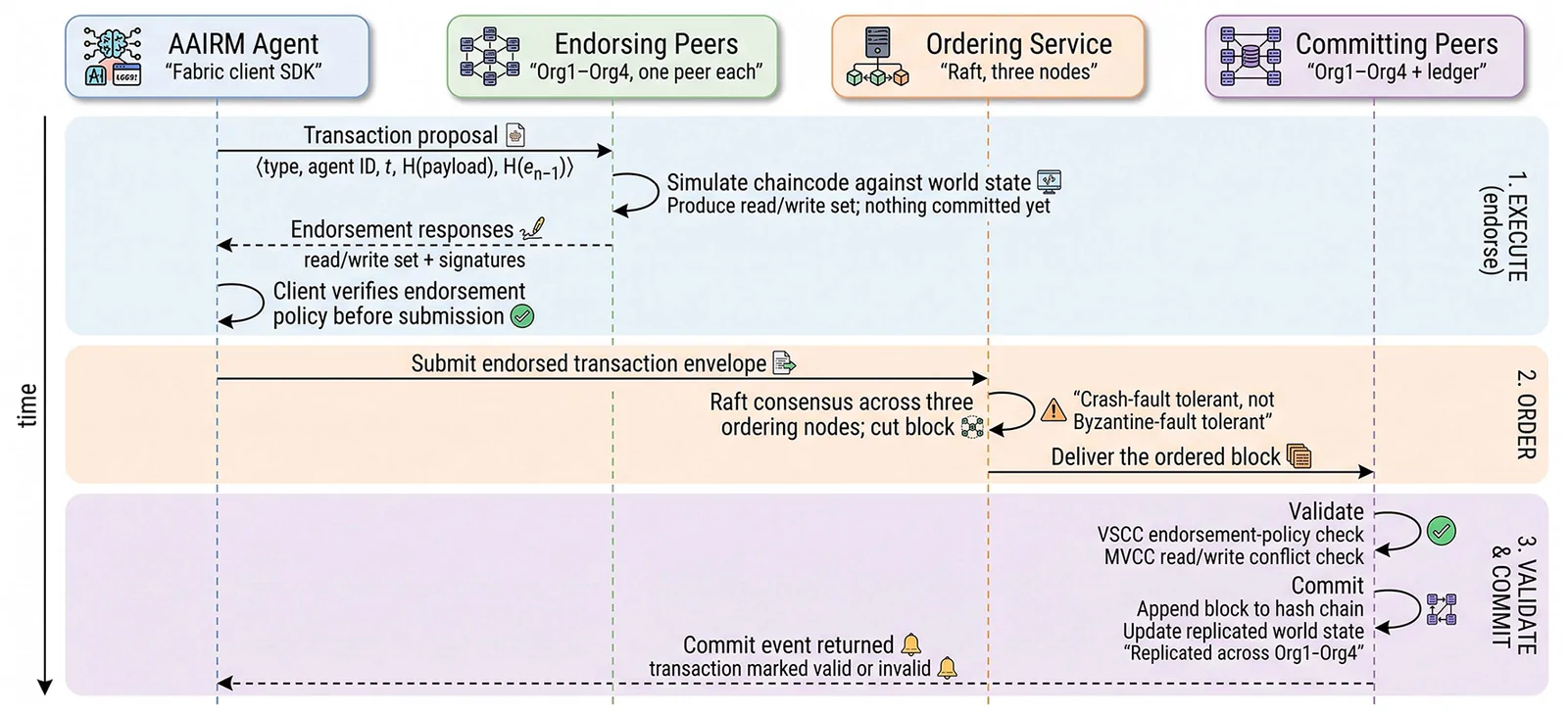
Transaction commit sequence on the four-organization permissioned Hyperledger Fabric testbed. The workflow follows the execute–order–validate architecture: transaction proposal and endorsement, ordering and block delivery, and validation and commit. Commit confirmation is returned asynchronously after ledger update.

**Table 11:** Blockchain trust ledger evaluation on the four-organization permissioned testbed.

Metric	BTL (permissioned ledger)	Centralized log
Mean commit latency (ms)	142	9
95th-percentile commit latency (ms)	287	21
Sustained throughput (tx/s)	310	>5,000
Audit query latency (ms)	38	9
Storage per event (KB)	1.9	0.6
Decision-cycle overhead (%)	6.6	< 0.5
Injected-mutation detection^†^ (500 cases)	500/500	0/500
Tamper evidence under insider mutation	Yes (multi-org consensus)	No (single trust domain)
Tamper evidence under majority collusion	No (see Table 2)	No

The centralized-log comparison uses the same workstation and an append-only PostgreSQL table. Latency, throughput, storage, and overhead rows are measured; the mutation-detection row is a mathematically guaranteed integration check, not an independent empirical security result, and it should not be read as evidence about the adversary classes in Table 2.

^†^Guaranteed by SHA-256 collision resistance; an integration check, not an independent empirical security measurement.

The verification protocol is exercised by a mutation-injection replay: 10,000 anchored events are replayed, and in 500 randomly selected cases one off-chain payload field is mutated after the fact. This validates the integration, confirming that digest computation, chain-continuity checks, and off-chain-to-on-chain correspondence are correctly assembled. It is not a security evaluation. The 100% detection rate is a mathematical consequence of SHA-256 collision resistance, so any correct implementation would produce it, and a failure would have indicated a wiring defect rather than a cryptographic one. The exercise covers exactly one row of Table 2 and says nothing about orderer collusion, chaincode exploits, ledger denial of service, or digest-store compromise. An equivalent mutation applied to a conventional centralized log is undetectable by construction since the log and its integrity reference share a trust domain. That architectural difference motivates the BTL design.

The comparison makes the governance trade-off explicit. The centralized log is faster and cheaper on every performance metric. The BTL's sole advantage is tamper evidence under insider or orchestrator compromise, and that is the property accountable procurement in a multi-party network requires. Deployments without multi-party trust or external audit requirements should prefer the centralized log. Those spanning retailer, suppliers, logistics, and auditors obtain accountability at a measured 6.6% latency overhead, subject to the boundary conditions of Section 3.4.1.

### 4.13 Computational cost and sensitivity analysis

Mean per-decision latency at 100 SKUs, covering the P1 query, C1 forecasting, C2 inference, C3 ranking, and A1 execution, is 3.2 s. Model inference accounts for roughly 1.8 s of that, across an average of 4.3 tool-routing calls, and at 500 SKUs the total rises to 8.7 s. Model API cost is approximately $0.003 per decision cycle at 100 SKUs, or roughly $1,100 annually at 1,000 daily decisions. Neither inference cost nor ledger anchoring binds at this scale, although tens of thousands of SKUs with real-time decisions would require batching, caching, or distilled models.

The cross-scale hyperparameter differences (Supplementary Table S2) follow standard scale-adaptation practice rather than tuning against the test metric. Warehouse capacity scales proportionally with SKU count while the purchasing budget scales sub-linearly, so the projection operators remain binding at both scales. A sensitivity sweep at 100 SKUs, 10 seeds per setting, varies the learning rate, clip ratio, entropy coefficient, and supplier weights across 22 configurations. Normalized cost stays within [0.861, 0.883], within 1.3% of the reported value, and no perturbation changes AAIRM's ordering relative to any baseline. The largest sensitivity is at the lowest entropy coefficient of 0.01, where two of 10 seeds converge to higher-cost local optima, indicating that exploration pressure rather than configuration defines the robustness margin.

### 4.14 External validation on public retail data

A purely synthetic evaluation cannot establish external validity. Real demand shows non-stationarity, promotional and calendar effects, long-tail intermittency, and supplier disruptions that an independent-shock generator does not capture. We therefore validate on the public M5 Forecasting Competition dataset (Makridakis et al., 2022b,a), the sole external corpus used here. Corporación Favorita (Corporación Favorita and Kaggle, 2017) is scoped as future work.

M5 records daily unit sales for 3,049 Walmart products across 10 stores in three US states, giving 30,490 item-store series over 1,913 training days plus the official 28-day horizon, with calendar, price, and promotion covariates. Supplementary Table S4 specifies the complete mapping onto the AAIRM environment. Three elements bear on interpretation. A SKU is one item-store series, and the evaluated assortment is the 1,200 highest-selling series, retaining real intermittency at a SKU count the policy was designed for. Perishability follows the mapped category rather than the raw label, and since M5 carries no expiry information any such treatment is imposed rather than observed. The 10 stores serve as federated clients, a non-synthetic non-IID partition. C1 is trained under the official Weighted Root Mean Squared Scaled Error (WRMSSE) and C2 in a lost-sales simulation on realized M5 demand, retrained for 100 episodes per seed across five seeds (42–46), against BL1, BL3, and BL5.

Cost parameters derive from the M5 price fields under stated assumptions: a wholesale proxy at 65% of mean sell price, a 20% annual holding-cost benchmark for grocery retail (Nahmias and Olsen, 2020), and a fixed stockout multiple on unit cost. M5 carries no supplier information whatever, so the supplier catalog is synthesized outright under a fixed seed, with all distributions given in Supplementary Table S4. These are assumptions grounded in standard retail benchmarks rather than measurements from supplier contracts, which bounds how far the M5 study validates the procurement layer as opposed to the ordering layer, a point Section 5.5 returns to.

Table 12 shows AAIRM attaining a 10.2% cost reduction over ROP–EOQ on real M5 demand (95% CI 8.0–12.4). That sits modestly below the 13.2% observed synthetically, as expected given greater volatility and intermittency, and both the small MAPPO advantage and the federated accuracy–cost trade-off persist. Replicating the governance and LLM ablation contrasts on M5 gives a coordination-and-governance contribution of 2.5 to 3.0 cost points, smaller and noisier than the 3.8-point synthetic figure, while the LLM orchestrator stays within the TOST equivalence margin, matching the synthetic null. The governance contribution therefore transfers to real demand alongside the aggregate cost advantage, with its magnitude attenuated. One retailer in one country is a narrow basis for that conclusion, and we treat it as evidence of transfer rather than of generality.

**Table 12:** External validation on the M5 dataset, five seeds (42–46), reported alongside the corresponding synthetic-evaluation figures.

Quantity	Synthetic (10 seeds)	M5 (5 seeds)
C1 forecast WRMSSE (centralized)	n/a	0.66 ± 0.04
FDL penalty vs. centralized (WAPE pts)	0.9 ± 0.1	1.65 ± 0.21
Cost reduction vs. ROP–EOQ (%)	13.2 ± 1.2	10.2 ± 1.8
Cost reduction vs. MAPPO (%)	1.9 ± 2.2	2.0 ± 2.4
Fill rate at default *w*_*p*_ (%)	92.3 ± 0.8	91.2 ± 1.1

WRMSSE is the official M5 accuracy metric; cost reduction is relative to ROP–EOQ on the M5 replenishment simulation. Full protocol is shown in Supplementary Table S4.

## 5 Discussion

### 5.1 Economic positioning and the nature of the contribution

Stated at a deployable operating point, the results build a consistent case for multi-agent, RL-driven inventory management under governance constraints. AAIRM retains a cost advantage at the measured 96.1% and 97.4% fill points that bracket the commercially standard 97% target (Section 4.10). The layered decomposition of Section 4.6 locates that advantage in the learned ordering policy first and the governance operator chain second, with LLM orchestration contributing effectively nothing. Against MAPPO, the picture is different in kind (Section 4.7). Once the same feasibility projection is granted to the comparator, the residual cost gap does not survive multiple-comparison correction, so the governance layer's defensible advantage there is hard constraint feasibility and an auditable record rather than a cost margin.

A fair appraisal requires stating precisely what AAIRM does and does not claim. It introduces no new learning algorithm, consensus protocol, or federated-aggregation rule. PPO (Schulman et al., 2017), Hyperledger Fabric (Androulaki et al., 2018), and FedProx (Li et al., 2020b) serve as established components. The contribution is empirical, systems-level, and governance-oriented: the component-resolved decomposition, the LLM null result, the downstream-cost characterization of federated learning together with its privacy boundary, the ledger integration with a stated adversary model, and the reward-hacking exposure and its correction, enumerated in Section 6. An honest characterization of how these components interact, including where they fail and how governance corrects them, is a different kind of contribution from algorithmic novelty, and we position it as such. Accordingly, AAIRM is best described as a multi-agent RL and governance framework rather than an “agentic AI” system centered on language-model inference. The model's role here is exception handling and natural-language interfacing, which may add value where communication protocols are heterogeneous, but which a structured simulation cannot assess.

### 5.2 Governance-aware deployment and human oversight

Four practical factors condition accountable deployment. Category-specific reward calibration is the most critical. Weight settings govern the cost–service trade-off per category, and miscalibrating them is an alignment failure with commercial and welfare consequences, as the dry-fruits case demonstrates. Organizations should treat these weights as a policy decision requiring explicit approval rather than as a technical hyperparameter. Human-oversight thresholds should be encoded at the chaincode layer as protocol-level guarantees, with one caveat that deployment planning should absorb. No human was in the loop during simulation, so the escalation threshold raised flags without blocking execution. This study therefore measures the cost of the mechanism at roughly zero and says nothing about its operational value. Ledger sizing follows from context: Multi-party consortia benefit from the four-organization deployment, single-retailer deployments may prefer a two-party ledger and still retain tamper evidence against insider modification, and any threat model including participant collusion requires the Byzantine-fault-tolerant configuration of Section 3.4.1, which we specify but do not evaluate. Federated learning follows data-sovereignty constraints: Single-site deployments should prefer centralized training, multi-banner networks should prefer FedProx, and any deployment making a privacy claim should add secure aggregation first.

None of this substitutes for organizational governance structures. Retailers should set escalation thresholds for high-value or unverified-supplier orders and should periodically audit the ledger for systematic bias or policy drift, particularly in high-spoilage categories. The 6.6% decision-cycle overhead (Section 4.12) is the measurable price: Under emerging governance frameworks (European Parliament and Council of the European Union, 2024), asserting correct behavior is not enough, and organizations must be able to demonstrate it.

### 5.3 Urban infrastructure and sustainability implications

In the urban context that this framework targets, retail supply chains are infrastructure as consequential as transit or utilities. Stockouts in grocery or frozen food carry welfare consequences, particularly in food-desert areas where alternatives are limited. AAIRM's measured 22.0% spoilage and 28.6% inventory reductions relative to ROP–EOQ are reductions in food waste whose environmental implications reach beyond a retailer's balance sheet. That magnitude is easy to state as arithmetic and hard to state as a validated result. Scaling it to avoided tonnage requires assumptions about throughput and baseline spoilage that we did not measure. Converting it to avoided emissions requires a life-cycle emissions factor that varies by an order of magnitude across product classes, and we do not estimate one. We therefore report no life-cycle figure.

The governance point is more robust than the sustainability arithmetic. AAIRM agents make consequential decisions about what is stocked, in what quantity, and from which suppliers, without a human in the loop for individual transactions. The ledger converts that otherwise invisible autonomy into a verifiable, challengeable record. Sustained throughput of 310 tx/s sits more than three orders of magnitude above expected production load at the evaluated scale, so the accountability infrastructure can absorb multi-store consolidation without becoming the bottleneck that tempts an operator to switch it off.

### 5.4 Labor and political economy: an architectural reading

Deploying autonomous procurement agents carries socioeconomic consequences that performance metrics do not capture. What follows is an architectural reading of which tasks the system automates and which it leaves to human judgment, offered as a hypothesis. It rests on no human-subjects data, no workforce measurements, and no labor-market analysis, and none of it is evidence about employment outcomes.

The labor footprint of autonomous replenishment falls most directly on routine procurement work: purchasing clerks, replenishment planners, inventory controllers, and forecasting analysts, whose core tasks AAIRM partially or fully automates. The scale of that exposure is contested along the lines set out in Section 2.7, where the displacement-vs.-augmentation question turns on whether the tasks that automation complements expand or contract (Frey and Osborne, 2017; Autor, 2015; Brynjolfsson and McAfee, 2014). The closest parallel to AAIRM's own domain is the algorithmic-management literature. Several of the management functions that Parent-Rocheleau and Parker (2022) catalog, notably monitoring and goal-setting, are instantiated directly by C2 and C5 for procurement rather than for personnel decisions. The collective-voice adaptations that Doellgast et al. (2023) document map onto the escalation-threshold and ledger-permissioning instruments proposed below.

Supplementary Table S5 gives the structured, task-level reading of AAIRM's likely effect on the urban retail roles it touches, separating tasks it automates outright from tasks it augments by surfacing decisions for human judgment. Routine ordering is the clearest displacement candidate. Replenishment planning, demand forecasting, supplier relationships, and category management lean toward augmentation, with category calibration and service-target policy remaining human governance decisions and the ledger supplying the record against which they are accountable. Warehouse and logistics labor is largely unaffected since AAIRM governs ordering rather than physical handling. Whether augmentation materializes as better jobs or as fewer jobs with degraded conditions is set by organizational and regulatory choices outside the architecture. The World Economic Forum projects large gross displacement alongside larger gross creation (World Economic Forum, 2023), wage and employment effects hinge on whether automation augments or replaces labor (Acemoglu and Restrepo, 2019), and the distributional literature (Eubanks, 2018; Crawford, 2021) indicates that the institutional settlement around the technology is the relevant unit of analysis.

One regional caveat is worth recording as a conditional rather than a finding. The automation-and-retail-work literature above is grounded in North American and Western European labor markets. Gulf Cooperation Council retail markets, the deployment context closest to this framework's origin, differ structurally in ways that would blunt the augmentation-leaning reading just given. Supplementary Section S3 sets out that argument in full. We state it as a conditional because we are aware of no published, architecture-linked empirical study of retail-automation labor effects specific to Gulf or wider Middle East and North Africa contexts, and we have conducted none.

#### 5.4.1 Policy levers for labor-sensitive governance

The governance substrate is this paper's concrete contribution and also the most direct lever available to policymakers engaging the labor question. Three design choices function as policy-relevant primitives rather than purely technical ones. First, escalation is configurable at deployment time. A labor-facing escalation policy could require human sign-off on decisions that would eliminate or materially restructure a role, rather than only on those above a monetary threshold. That is a parameter a regulator or works council can mandate, not a research question requiring new architecture. Second, the ledger's audit trail is by construction available beyond the deploying retailer, so extending read access to labor regulators, worker representatives, or independent auditors is a permissioning decision on an existing channel, echoing the collective-voice mechanisms Doellgast et al. (2023) document in a non-algorithmic setting. Third, the per-category service and stockout-penalty weights that the Pareto analysis exposes as tunable also determine which neighborhoods experience degraded fill rates under cost-minimizing configurations. Making those weights, and any deviation from a uniform-service default, part of a disclosed governance record is a concrete instrument for the equity concerns that Eubanks (2018) raises. These are not substitutes for organizational governance structures but the technical substrate such structures depend on. Worker surveys, occupational task-content studies, and comparative regional case studies are the work that would test the reading above.

### 5.5 Limitations and threats to validity

The most consequential limitation is the evidentiary one of Section 1.1: The primary and scalability experiments use synthetic demand, and the single external corpus represents one retailer in one country. Because M5's cost parameters are benchmark-grounded assumptions, its supplier catalog is synthesized outright, and its perishability is imposed rather than observed, the M5 study validates the ordering and forecasting layers considerably more strongly than the procurement and negotiation layers. The Corporación Favorita dataset remains a direction for future validation alongside live deployment.

Three methodological asymmetries deserve explicit statement. First, baselines were held at conventional parameter settings rather than tuned to their own cost-optimal operating points (Section 4.3). Retuning the ROP–EOQ safety factor would narrow the headline gap measured at AAIRM's leanest point, which is why we regard the matched-service comparisons as decisive. Second, we did not evaluate constrained-RL comparators, so the argument of Section 4.7.1 for structural over Lagrangian enforcement rests on the character of the constraints and on the governance properties of an inspectable rule, not on a measured comparison. Third, the layered governance decomposition adds one mechanism at a time in a fixed order, so the reported increments are order-dependent and interaction effects between mechanisms are not identified.

On accountability, the tamper-evidence result validates the integration rather than resilience to a capable adversary. Section 3.4.1 states the boundary. On privacy, the federated results quantify the accuracy and cost of decentralization but establish no formal guarantee, since neither secure aggregation nor differentially private training was applied. The heterogeneity regime evaluated (β = 0.5) is moderate, so the penalty of Section 4.11 is a lower bound on the cost of a formally private deployment. Statistical power is limited at 500-SKU scale, where five seeds support only indicative variance estimates and extension to 10 seeds remains a validation priority. The Agent Health Monitor, the Reputation and Scoring Engine, and the Zero-Knowledge Proof layer remain architecturally specified with no evaluation. Comparison on established external benchmarks (Oroojlooyjadid et al., 2022; Gijsbrechts et al., 2022; De Moor et al., 2022) remains a priority. Finally, the labor and organizational implications require interdisciplinary investigation beyond the scope of a technical contribution, which we regard as a genuine boundary rather than a gap to be papered over with architectural speculation.

## 6 Conclusion

As AI systems take on operational authority over urban retail infrastructure, governance becomes as essential a design criterion as efficiency. AAIRM addresses that dual mandate, achieving measurable efficiency gains in simulation while supplying the accountability infrastructure that trustworthy autonomous systems require.

This study makes six contributions.

- **A governance-aware architecture whose constraints hold by construction**. A Perception–Conceptualization–Action pipeline spans forecasting, constrained reorder optimization, supplier selection, negotiation, and execution. The Governance Agent (C5) enforces budget, capacity, shelf-life, escalation, and supplier-concentration rules structurally rather than through a learned penalty and anchors every decision it clears in a tamper-evident audit trail (Section 3.2.4).
- **A component-resolved account of where the advantage comes from**. The layered decomposition splits the coordination-and-governance block into feasibility projection, escalation and anomaly holds, cross-category policy rules, supplier ranking, and negotiation and shows analytically that ledger anchoring contributes nothing to cost and everything to auditability. Strengthened RL baselines, a feasibility-matched comparator, and M5 external validation keep that attribution from resting on weak comparisons.
- **A transparent null result on LLM orchestration**. In structured, deterministic procurement, the LLM-mediated orchestrator adds no statistically significant economic value over deterministic tool routing [Δ = 0.6 points, 95% CI (−0.9, 2.1), *p* = 0.38], and two one-sided tests confirm practical equivalence within a ±2-point margin. We report it as a first-class finding, scoped to structured environments.
- **A trust ledger with an explicit adversary model**. A four-organization Hyperledger Fabric ledger anchors proposals, verdicts, terms, and execution confirmations as hash-chained transactions at 6.6% decision-cycle overhead. A 500-case mutation replay validates the integration, and we state which adversaries the design withstands and which it does not, including that crash-fault-tolerant ordering gives no protection against a colluding majority.
- **Federated demand learning with an explicit privacy boundary**. FedAvg and FedProx train the forecaster across stores without centralizing raw sales data, at a cost of 1.3 points for FedProx and 1.8 for FedAvg while retaining an 11%–12% advantage over ROP–EOQ. We separate what withholding raw data achieves from what it does not and identify where secure aggregation and differential privacy become necessary.
- **A reward-hacking failure and its governance correction**. At 500-SKU scale, default penalty weights drive a 31.54% stockout rate in dry fruits. We analyze this as an alignment failure in which the agent exploits a miscalibrated objective (Amodei et al., 2016; Skalse et al., 2022; Pan et al., 2022) and demonstrate category-specific governance constraints as the correction.

Taken together, these results establish that governance-aware autonomous procurement is operationally viable at deployable service levels. AAIRM holds an 8.2-point cost advantage over ROP–EOQ at 96.1% fill and 4.9 points at 97.4%, a 13.2% reduction at the leanest operating point (95% CI 12.2–14.2), and 10.2% on real M5 demand. Hash-chain audit costs 6.6% overhead, and federated training lands within 0.9 WAPE points of centralized at a 1.3-point cost penalty. Against a strong multi-agent RL baseline, the governance layer's decisive contribution is hard feasibility and an auditable record rather than a cost margin, since once the same projection is granted to MAPPO the residual gap does not survive correction. Clarifying where autonomous intelligence adds value, and where it does not, matters for responsible deployment as much as for technical positioning.

The dry-fruits failure is the most instructive of these findings. RL agents in commercial settings can exploit formally specified reward structures in ways that satisfy the mathematical objective while violating organizational intent. Encoding the correction at the chaincode layer makes it auditable, externally configurable, and verifiable by any authorized party. That is the operational meaning of bounded autonomy: not the absence of autonomous decision-making, but governance infrastructure that makes autonomy correctable, transparent, and accountable. The same framing requires naming what the infrastructure does not do, which is why this study states its adversary model and its privacy boundary rather than leaving either implicit.

Four directions should drive future work. The first is deployment validation across diverse retail geographies, with Corporación Favorita an intermediate stepping stone. The second is security and privacy hardening: Byzantine-fault-tolerant ordering, secure aggregation, differentially private training, adversarial evaluation of prompt injection, and formal verification of chaincode-encoded governance constraints. The third is constrained-RL comparators and scalability to commercial SKU counts. The fourth is interdisciplinary study of the labor consequences, including worker surveys and comparative regional case studies grounded in Gulf and wider Middle East and North Africa retail labor markets, where the human stakes are concentrated and least studied.

## Data Availability

The original contributions presented in the study are included in the article/Supplementary material, further inquiries can be directed to the corresponding author.
