# Supplementary material for: Governance-aware autonomous retail coordination in artificial intelligence cities using multi-agent reinforcement learning, blockchain accountability, and federated learning
Source: Front Artif Intell. 2026 Oct 7;9:1942376. doi: 10.3389/frai.2026.1942376 (PMC13683724; doi:10.3389/frai.2026.1942376)
Supplement: Supplementary file 1 [file Data_Sheet_1.pdf]

Governance-Aware Autonomous Retail Coordination in Artificial Intelligence Cities Using Multi-Agent Reinforcement Learning, Blockchain Accountability, and Federated Learning

This Supplementary Material accompanies the main manuscript. It contains four supporting figures, six supporting tables, and three extended discussion sections, comprising the notation, configuration, protocol, per-category detail, and socio-technical analysis moved out of the main text to satisfy the target journal's word limit for Original Research articles. The main text refers to these items by their S-numbers: Table S2 gives the full experimental configuration, Table S4 the complete external-validation protocol on the M5 dataset, Table S5 the task-level labor reading, Section S3 the regional labor-market argument summarized in the main text's discussion of labor and political economy, Section S4 the constrained-RL design alternatives to the governance layer's projection operators, Section S5 the full decision-process specification of the Reorder Optimization Agent, and Table S6 the evaluation comparability protocol across the eight policies.

S1 SUPPLEMENTARY FIGURES

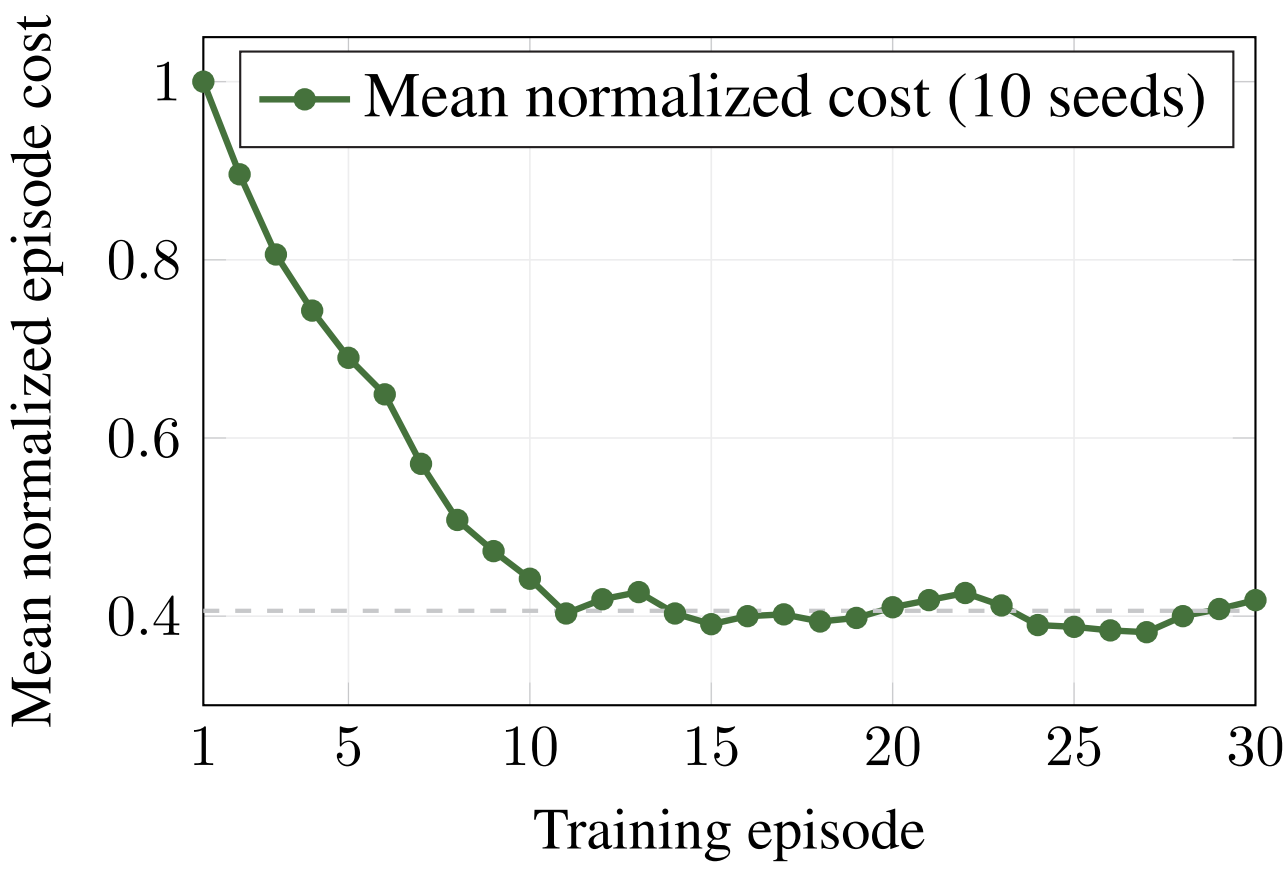

Figure S1. Mean normalized per-episode cost of the Reorder Optimization Agent (C2) over the first 30 of 200 training episodes across 10 seeds in the 100-SKU experiment. The dashed line indicates the mean stabilized cost (≈ 0.41).

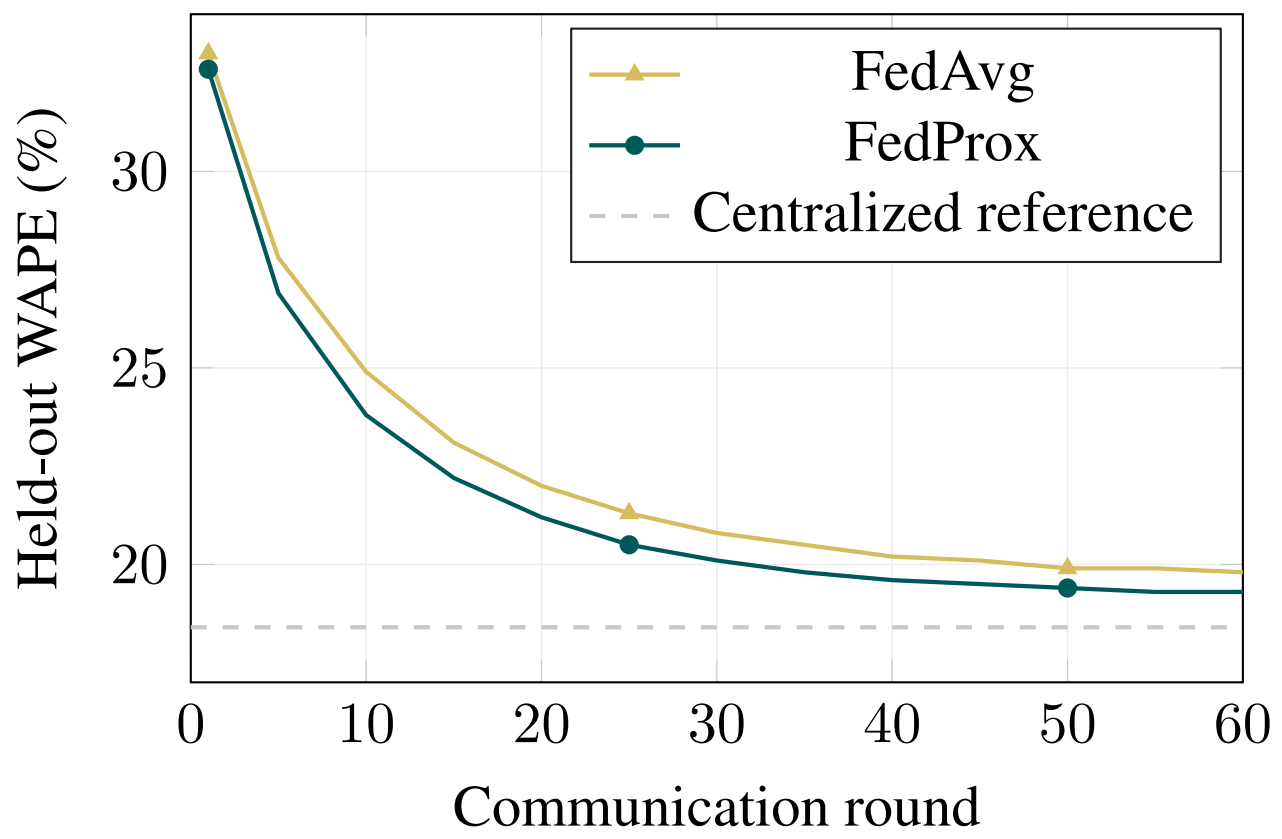

Figure S2. Convergence of federated demand learning over 60 communication rounds across eight non-IID stores. FedAvg and FedProx are evaluated at Dirichlet concentration $\beta = 0.5$; the dashed line denotes the centralized reference.

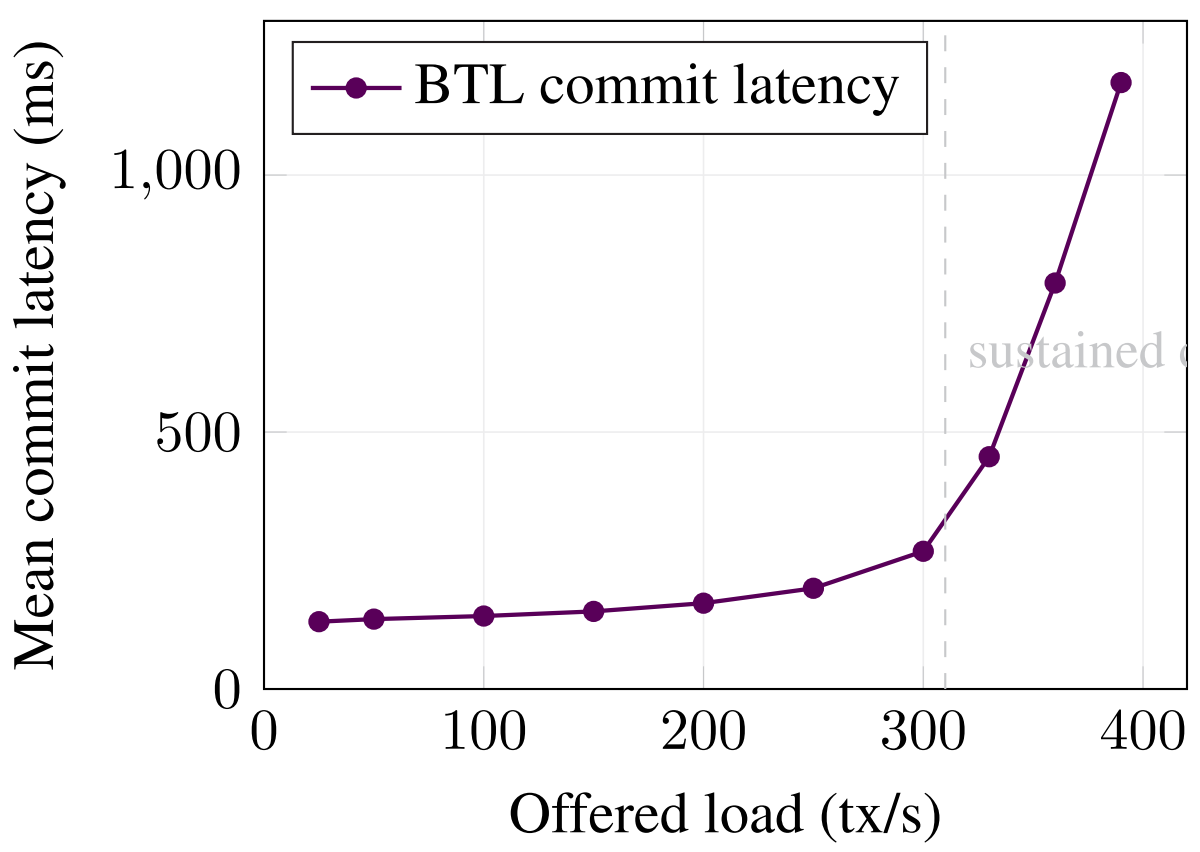

Figure S3. Blockchain Trust Ledger commit latency versus offered anchoring load on the four-organization testbed. The dashed line marks the sustained capacity of approximately 310 tx/s.

S2 SUPPLEMENTARY TABLES

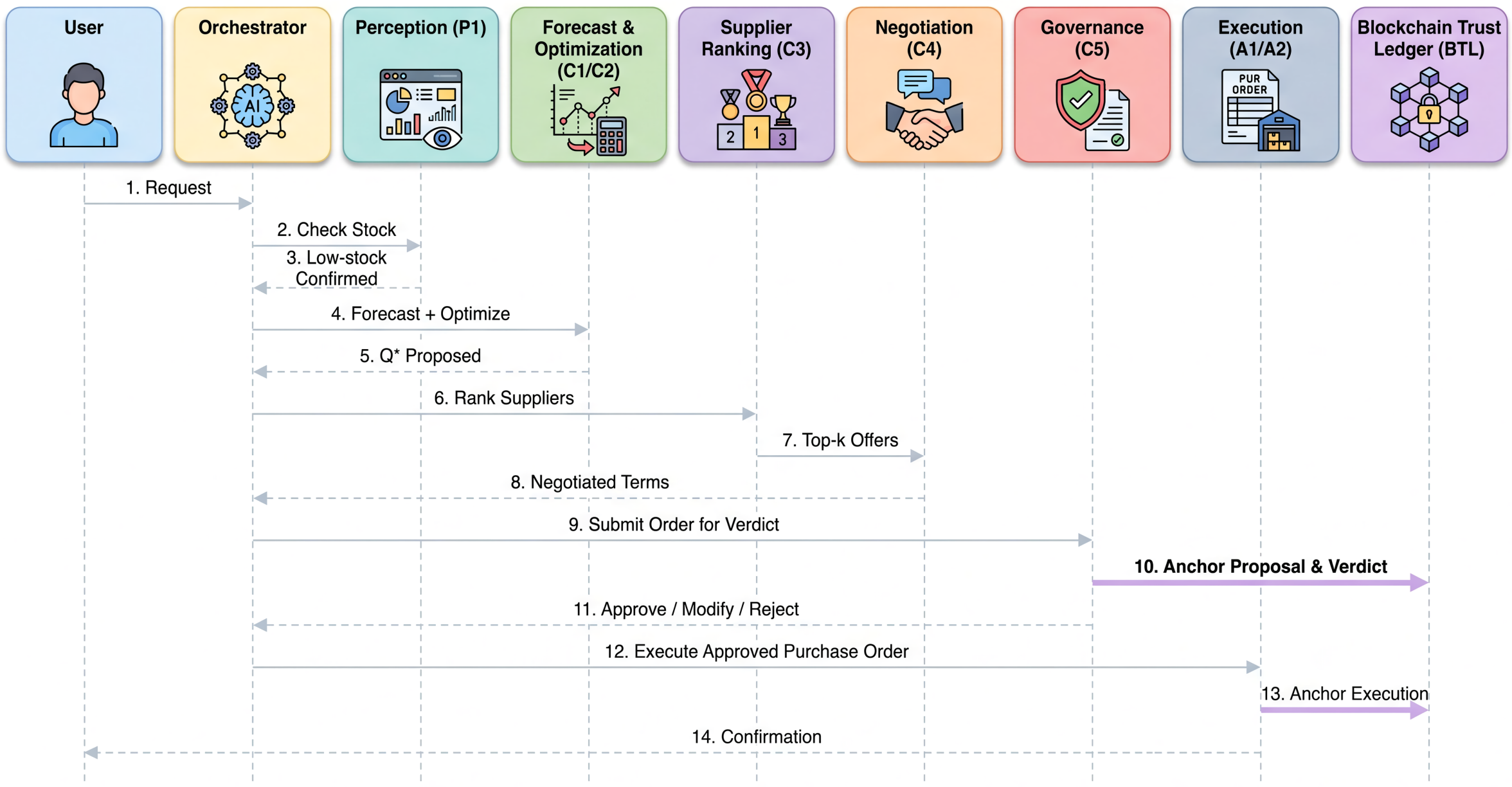

Figure S4. AAIRM replenishment workflow from user request to execution and ledger anchoring. The sequence covers perception, forecasting and optimization, supplier ranking and negotiation, governance, execution, and blockchain-based anchoring.

Table S1. Principal notation used throughout the framework and evaluation. The three quantities most prone to confusion are listed first: the demand-distribution mean, the policy-network action mean, and the FedProx proximal coefficient are kept notationally distinct.

Symbol	Meaning
$\mu_D,\ \sigma_D$	Mean and standard deviation of per-period demand
$\mu^{\pi}_{i,t},\ \varsigma_{i,t}$	Mean and standard deviation of the policy's order action for SKU i at epoch t
λ	FedProx proximal-term coefficient
$I_{i,t},\ \rho_{i,t}$	On-hand and in-transit (pipeline) inventory for SKU i
$\tau_{i,t}$	Remaining shelf life for SKU i
$\bar{d}_i$	Mean daily demand for SKU i, used by the shelf-life operator
$\hat{y}_{i,t+h},\ \hat{\sigma}_{i,t}$	Demand forecast and forecast-uncertainty estimate
$Q_{i,t},\ Q_i^{\max}$	Order quantity and its per-SKU upper bound
$H,\ \gamma$	Planning horizon and discount factor
$B,\ B_c$	Total purchasing budget and its per-category allocation, $\sum_c B_c = B$
$V,\ V_z$	Warehouse-capacity bound and its per-storage-zone allocation, $\sum_z V_z = V$
$c_j,\ v_j$	Unit cost and unit volume of SKU j
z	Safety factor for the ROP–EOQ baseline
$w_p,\ w_p^{\mathrm{DF}}$	Stockout penalty weight (general; dry-fruits category)
$\alpha_1, \ldots, \alpha_4$	Supplier-scoring weights
$\Pi_{\mathrm{hold}},\ \Pi_{\mathrm{bud}}$	Governance operators: anomaly hold; category-budget projection
$\Pi_{\mathrm{cap}},\ \Pi_{\mathrm{life}}$	Governance operators: storage-zone projection; shelf-life alignment
$\Theta,\ \kappa,\ \psi$	Human-approval threshold; shelf-life safety margin; supplier-concentration limit
$\mathcal{A}_t$	Set of SKUs flagged by the anomaly detector (P5) at cycle t
$\theta,\ F_k(\theta)$	Forecaster parameters and client-k local empirical risk
$K,\ E,\ U$	Number of federated stores, local epochs per round, communication rounds
β	Dirichlet concentration controlling inter-store heterogeneity
$V_\phi(s)$	Critic state-value function

S3 EXTENDED SOCIO-TECHNICAL ANALYSIS: REGIONAL LABOR-MARKET CONDITIONALITY

The main text gives an architectural reading of which procurement tasks AAIRM automates and which it leaves to human judgment, and summarizes it in Supplementary Table S5. That reading leans toward augmentation for planning, forecasting, supplier-relationship, and category-management roles. This section records the regional condition under which that lean would not hold, and states it as a conditional rather than a finding.

Most of the automation-and-retail-work literature that the main text cites is grounded in North American and Western European labor markets, where displaced workers typically retain domestic mobility and access to public retraining infrastructure. Gulf Cooperation Council retail markets, the deployment context closest to this framework's origin, differ structurally: frontline procurement, stocking, and warehouse-adjacent roles are disproportionately staffed by migrant labor under visa-sponsorship arrangements in several jurisdictions, which constrains workers' ability to change employers or to convert augmented roles into upskilled positions on the terms available to citizen employees in higher-tier planning roles.

If that structural difference holds, the augmentation-leaning reassignment described in the main text would not translate into equivalent bargaining power across the workforce that the system touches. Salaried planning, supplier-relationship, and category-management roles would be more likely to capture the gains than routine-ordering roles, and the distributional consequence of deployment would therefore depend on the labor-market institutions of the deployment context rather than on the architecture alone. The policy levers proposed in the main text, namely labor-facing escalation thresholds, extended ledger read access for worker representatives or regulators, and disclosure of per-category service weights, are correspondingly more consequential in this setting than in the markets that the cited literature describes.

We state all of this as a conditional. We are not aware of published, architecture-linked empirical studies of retail-automation labor effects specific to Gulf or wider Middle East and North Africa contexts, and we have conducted none. This section is an illustration of why deployment context matters to the reading in Supplementary Table S5, not a finding about Gulf labor markets, and it rests on no human-subjects data, no workforce measurements, and no labor-market survey.

S4 CONSTRAINED-RL DESIGN ALTERNATIVES TO FEASIBILITY PROJECTION

The main text argues that AAIRM's governance layer should enforce the budget and capacity ceilings of the reorder optimization problem structurally, by projection, rather than through a Lagrangian penalty, and gives two reasons: the constraints are hard per-period ceilings rather than expectations, and a projection operator is inspectable and auditable in a way a learned multiplier is not. This section records the remaining trade-offs, which run partly against that choice and are worth stating explicitly.

Expected cost penalty of truncation

A Lagrangian policy learns to anticipate the constraint through a shaped objective rather than being truncated at execution, so it should waste less of its action range on proposals that will subsequently be clipped. We would therefore expect a well-tuned PPO-Lagrangian or MAPPO-Lagrange policy to recover part of the 1.1-point normalized-cost gap the main text reports between AAIRM and BL6 (MAPPO with post-hoc projection). AAIRM already captures some of that benefit, because the Reorder Optimization Agent trains against the projected outcome rather than the raw proposal; this is the mechanism by which in-loop projection outperforms post-hoc projection rather than merely matching it. How much of the residual gap a Lagrangian formulation would close is an empirical question this study does not answer.

Tuning burden

Lagrangian methods introduce a multiplier learning rate that is known to induce oscillation in constraint satisfaction unless explicitly controlled, for example by a PID scheme on the multiplier. That is a tuning burden a projection operator does not carry, and it interacts with the per-category reward weights that the main text already identifies as requiring explicit organizational approval rather than technical tuning. A deployment adopting a Lagrangian formulation would therefore acquire a second class of safety-relevant hyperparameter alongside the first.

Action-space embedding

Embedding the budget directly in the action space, for instance by parameterizing orders as a simplex allocation of the available budget, would guarantee budget feasibility by construction with no projection step at all. Two properties argue against it here. First, it couples all SKUs within a single action distribution, breaking the per-SKU factorization that keeps the policy's parameter count independent of SKU count and that enables the transfer from 100 to 500 SKUs reported in the main text. Second, it addresses only one of the four constraint families the Governance Agent enforces, leaving storage-zone capacity, shelf-life alignment, and anomaly holds to be enforced separately, so a projection stage would be required regardless.

Status

A PPO-Lagrangian and MAPPO-Lagrange comparator suite remains a priority for future work. We expect it to sharpen rather than overturn the reading in the main text: the guarantee-class and auditability arguments are structural and do not depend on the measured cost difference, whereas the cost argument is the one a comparator suite could revise.

S5 DECISION-PROCESS SPECIFICATION OF THE REORDER OPTIMIZATION AGENT

The main text states the constrained optimization problem the Reorder Optimization Agent (C2) solves, the two design properties that matter downstream (per-SKU factorization and in-loop projection), and the reward. This section gives the state, action, and network specification in full. Symbols follow Supplementary Table S1 and numerical settings follow Supplementary Table S2.

State

The per-SKU state $s_{i,t}$ is the concatenation of:

- on-hand inventory $I_{i,t}$ and in-transit (pipeline) inventory $\rho_{i,t}$, both normalized by mean demand;
- the forecast vector $(\hat{y}_{i,t+1}, \ldots, \hat{y}_{i,t+H})$ over the planning horizon H, and its uncertainty estimate $\hat{\sigma}_{i,t}$;
- remaining shelf life $\tau_{i,t}$, together with recent realized spoilage and stockout fractions;
- the lead-time estimate $\hat{L}_{i,t}$ and the reliability of the currently preferred supplier.

A shared global context g_t is appended to every SKU's state and comprises the budget-utilization ratio $\sum_j c_j Q_j / B$, the capacity-utilization ratio $\sum_j v_j Q_j / V$, and a category indicator. The full state is therefore $s_t = \big(\{s_{i,t}\}_i,\, g_t\big)$.

The policy is factored per SKU: the same network is applied to every SKU with g_t broadcast to each. This keeps the parameter count independent of the number of SKUs, and it is the mechanism by which a policy trained at 100 SKUs transfers to the 500-SKU setting without architectural change. It also means that cross-SKU coupling reaches the policy only through g_t, which is why the joint budget and capacity constraints require the explicit projection step described in the main text rather than being resolvable within the policy itself.

Action

The action $a_{i,t}$ is a squashed Gaussian. The actor emits a mean and log standard deviation $(\mu^{\pi}_{i,t}, \log \varsigma_{i,t})$, a sample $\tilde{a}_{i,t} \sim \mathcal{N}(\mu^{\pi}_{i,t}, \varsigma^2_{i,t})$ is drawn, and the proposed order quantity is

$$Q_{i,t} = Q_i^{\max} \cdot \tfrac{1}{2}\big(1 + \tanh \tilde{a}_{i,t}\big) \in [0, Q_i^{\max}], \quad \text{(S1)}$$

where $Q_i^{\max}$ is a per-SKU cap derived from the constrained optimization problem of the main text. The proposal is then passed through the Governance Agent's projection operators before application, and C2 trains against the projected outcome rather than the raw proposal.

Reward and networks

The per-epoch reward is $r(s_t, a_t) = -\sum_i C_i(Q_{i,t})$, the negative of the single-period expected cost summed over SKUs, so the discounted-return objective equals cumulative discounted negative cost. The actor and critic are separate three-layer multilayer perceptrons (256–256–128 units, $\tanh$ activations); the critic outputs a scalar state value $V_\phi(s_t)$ and is trained on the temporal-difference error given in the main text. Policy optimization uses PPO with generalized advantage estimation; learning rate, clip ratio, entropy and value coefficients, discount factor, rollout length, minibatch size, and epochs per update are listed for both scales in Supplementary Table S2.

Table S2. Configuration of the primary (100-SKU) and scalability (500-SKU) inventory simulation experiments.

Parameter	Primary (100-SKU)	Scalability (500-SKU)
Simulation and Evaluation		
SKU count	100	500
Category composition	Grocery 30, Frozen Food 20, Apparel 15, Cosmetics 20, Dry Fruits 15	100 SKUs/category (5 categories)
Simulation / evaluation horizon (days)	730 / 365	730 / 365
Steps per episode / training episodes	365 / 200	365 / 200
Environment steps per seed	$\approx 7.3 \times 10^4$	$\approx 7.3 \times 10^4$
Random seeds	10 (42–51)	5 (42–46)
Suppliers per SKU	3–5	3–5
Lead time by category (mean/SD [min,max], days)	G 2/0.5 [1,4]; F 2/0.5 [1,3]; A 5/1.0 [3,10]; C 4/1.0 [2,7]; D 3/0.5 [2,5]	Same as primary
Cost and Reward Weights		
Holding / spoilage cost weights	1.0 / 1.0	1.0 / 1.0
Stockout penalty w_p	1.2	1.2
Inventory cap penalty / horizon	0.5 / 21 days	0.5 / 21 days
Annual holding-cost rate	0.25	0.25
Stockout penalty multiplier	3.0	3.0
Shelf-life scale / expiry multiplier	0.5 / 1.5	0.5 / 1.5
Forecaster (C1)		
Architecture	LSTM encoder–decoder	LSTM encoder–decoder
Context / forecast horizon (days)	60 / 7	60 / 7
Hidden size / dropout	128 / 0.1	128 / 0.1
Batch / max epochs / learning rate	64 / 50 / 1×10^{-3}	64 / 50 / 1×10^{-3}
Loss	Mean squared error	Mean squared error
LLM Specification		
Model / provider	GPT-4o-mini (OpenAI)	GPT-4o-mini (OpenAI)
API model version	`gpt-4o-mini-2024-07-18`	`gpt-4o-mini-2024-07-18`
Temperature / `max_tokens`	0.1 / 1024	0.1 / 1024
PPO Hyperparameters		
Learning rate	2×10^{-4}	1×10^{-4}
Clip ratio / entropy coefficient	0.2 / 0.02	0.2 / 0.025
Value coefficient / discount factor	0.5 / 0.99	0.5 / 0.995
Rollout / minibatch / epochs	2048 / 64 / 10	2048 / 64 / 10
Actor / critic architecture	256–256–128 MLP, $\tanh$	256–256–128 MLP, $\tanh$
Gradient-boosting library	XGBoost	XGBoost
BL2/BL3 `n_estimators`	200	300
Supplier Scoring Weights		
α_1 / α_2 (unit cost / lead time)	0.45 / 0.25	0.40 / 0.25
α_3 / α_4 (reliability / MOQ)	0.20 / 0.10	0.25 / 0.10
Governance Agent (C5)		
Purchasing budget B	1.2×10^5	5.5×10^5
Warehouse capacity V	9.0×10^3	4.5×10^4
Frozen / ambient capacity	2.0×10^3 / 7.0×10^3	1.0×10^4 / 3.5×10^4
Category budget allocation B_c	Uniform, $B/5$	Uniform, $B/5$
Human-approval threshold Θ	$50,000	$50,000
Shelf-life margin κ / concentration limit ψ	0.80 / 0.60	0.80 / 0.60
Federated Demand Learning		
Clients K / local epochs E / rounds U	8 / 2 / 60	Not evaluated at this scale
Dirichlet concentration β	0.5	—
FedProx coefficient λ	0.01	—

Table S3. Per-category performance comparison at 100-SKU scale, averaged over ten seed-matched trajectories (seeds 42–51), for all four fully evaluated policies. Stockout rate and fill rate are percentages of demand; average inventory is in units per SKU; spoilage is a percentage of procured units. N/A denotes non-perishable categories with zero spoilage by construction. Category SKU composition is given in Supplementary Table S2.

Category	Policy	Stockout (%)	Fill rate (%)	Avg. inv.	Spoilage (%)
Grocery (30 SKUs)	BL1	1.16 ± 0.44	98.84 ± 0.44	6.99 ± 0.39	N/A
	BL2	9.32 ± 5.93	90.68 ± 5.93	3.74 ± 0.66	N/A
	BL3	3.50 ± 0.71	96.50 ± 0.71	5.90 ± 0.33	N/A
	AAIRM	9.13 ± 1.29	90.87 ± 1.29	4.40 ± 0.32	N/A
Frozen Food (20 SKUs)	BL1	1.18 ± 0.68	98.82 ± 0.68	8.26 ± 0.40	6.93 ± 1.04
	BL2	$1.14 \pm 2.62^{\dagger}$	$98.86 \pm 2.62^{\dagger}$	10.23 ± 2.85	8.87 ± 3.00
	BL3	2.15 ± 0.56	97.85 ± 0.56	6.80 ± 0.39	6.00 ± 0.81
	AAIRM	6.62 ± 1.59	93.38 ± 1.59	5.78 ± 0.31	5.18 ± 0.82
Apparel (15 SKUs)	BL1	0.35 ± 0.30	99.65 ± 0.30	11.15 ± 0.48	N/A
	BL2	0.00 ± 0.00	100.00 ± 0.00	25.60 ± 8.51	N/A
	BL3	0.90 ± 0.35	99.10 ± 0.35	8.80 ± 0.44	N/A
	AAIRM	3.54 ± 1.14	96.46 ± 1.14	8.44 ± 0.23	N/A
Cosmetics (20 SKUs)	BL1	1.06 ± 0.66	98.94 ± 0.66	7.47 ± 0.57	5.30 ± 0.79
	BL2	0.61 ± 1.21	99.39 ± 1.21	10.88 ± 3.34	7.87 ± 2.42
	BL3	2.70 ± 0.67	97.30 ± 0.67	6.10 ± 0.36	4.75 ± 0.62
	AAIRM	7.41 ± 2.61	92.59 ± 2.61	5.16 ± 0.37	3.92 ± 0.34
Dry Fruits (15 SKUs)	BL1	1.51 ± 0.76	98.49 ± 0.76	6.29 ± 0.62	15.20 ± 1.84
	BL2	4.70 ± 4.33	95.30 ± 4.33	4.83 ± 1.08	11.62 ± 2.92
	BL3	4.95 ± 1.24	95.05 ± 1.24	4.55 ± 0.51	16.30 ± 1.95
	AAIRM	7.75 ± 2.44	92.25 ± 2.44	4.50 ± 0.33	12.03 ± 1.32

† The Frozen Food BL2 stockout mean of 1.14% has a cross-seed standard deviation of 2.62 percentage points, exceeding the mean, which indicates a strongly right-skewed distribution driven by at least one high-stockout seed. The mean should not be read as a central tendency for this cell.

Table S4. Complete protocol for the external validation on the M5 Forecasting Competition dataset. All mappings and derived parameters are implemented in the released dataset adapter; parameters marked as assumptions are not measured from the data and bound how strongly the M5 study validates the procurement layer as opposed to the ordering and forecasting layers.

**Protocol element**	**Specification**
Source files	`sales_train_validation.csv`, `calendar.csv`, `sell_prices.csv`
Dataset scope	3,049 items × 10 stores × 3 US states = 30,490 item-store series; 1,913 training days plus the official 28-day evaluation horizon
SKU definition	One item-store series, identified as `item_id` ⊕ `store_id`
Evaluated assortment	The 1,200 series with the highest cumulative unit sales
Category mapping	FOODS_1, FOODS_2 → grocery; FOODS_3 → frozen food; HOBBIES_1 → apparel; HOUSEHOLD_1 → cosmetics; HOBBIES_2, HOUSEHOLD_2 → dry fruits; unmapped departments default to grocery
Perishability treatment (assumption)	Assigned by mapped category, not by the raw Walmart label: frozen food, cosmetics, and dry fruits are perishable and draw shelf lives from the same truncated exponential (scale 0.5, expiry-rate multiplier 1.5) used in the synthetic study; grocery and apparel take $\delta_i = 0$. M5 contains no expiry data, so any perishability treatment is imposed rather than observed.
Unit cost (assumption)	Wholesale proxy at $0.65\,\pi_i$, where π_i is the mean sell price of item i across the price panel
Holding cost (assumption)	$h_i = 0.20\,\pi_i/365$ per unit per day, a 20% annual benchmark for grocery retail
Stockout penalty (assumption)	Fixed multiple of unit cost, at the same $3.0\times$ multiplier used in the synthetic study
Unit volume (assumption)	0.05 volume units per unit, uniform across SKUs
Supplier catalog (synthesized)	M5 carries no supplier data. Three to five suppliers per SKU generated under a fixed seed, with unit cost $\sim \mathcal{U}[0.90, 1.30]\times$ wholesale proxy, lead-time mean $\sim \mathcal{U}[2, 10]$ days, lead-time SD $\sim \mathcal{U}[0.3, 2.0]$ days, reliability $\sim \mathcal{U}[0.70, 0.99]$, and MOQ drawn from $\{10, 20, 50, 100\}$
Exogenous covariates	Day of week, day of year, holiday flag derived from the two event-name fields, and the SNAP benefit flag
Federated partition	The ten M5 stores serve as clients $k = 1, \ldots, 10$, a non-synthetic non-IID partition
Forecaster (C1)	Temporal Fusion Transformer, 90-day context, 28-day horizon, pinball loss, quantiles $\{0.1, 0.5, 0.9\}$; accuracy reported as the official WRMSSE
Policy (C2)	Lost-sales replenishment simulation on realized M5 demand, retrained for 100 episodes per seed with the PPO configuration of Supplementary Table S2
Train/test split	80/20 by time
Seeds	5 (42–46)
Comparators	BL1 (ROP–EOQ), BL3 (ML + Adaptive), BL5 (MAPPO)

Table S5. Structured augmentation-versus-displacement reading of AAIRM's effect on principal urban retail roles, referenced from the main text's socio-technical discussion. This is an architectural analysis of task exposure derived from which pipeline stages the system automates. It is not a measured workforce outcome, rests on no human-subjects or labor-market data, and is offered as a hypothesis for organizational and labor-economics research rather than as evidence.

Role	Dominant tendency	Mediating mechanism and residual human task
Purchasing clerk, routine ordering	Displacement of routine ordering	Per-order automation by C2 removes manual reorder entry; residual work shifts to exception handling surfaced by the orchestrator and to the anomaly holds raised by C5.
Replenishment and inventory planner	Mixed, leaning augmentation	C2 sets quantities, but category calibration and service-target policy, such as the dry-fruits penalty weight, remain human governance decisions with commercial consequences.
Demand-forecasting analyst	Augmentation	C1 and the federated layer automate base forecasting; analysts move to model oversight, drift detection, and promotional-event judgment.
Supplier-relationship manager	Augmentation	C3 ranks suppliers and C4 handles routine terms, but onboarding, dispute resolution, and relationship management remain human, supported by the ledger record.
Store and category manager	Augmentation	Escalation thresholds in C5 route high-stakes decisions to managers; the ledger supplies the audit record against which those decisions are accountable.
Warehouse and logistics labor	Largely unaffected	AAIRM governs ordering rather than physical handling; downstream effects are indirect, via order volume and timing.

Table S6. Evaluation comparability protocol. All eight policies share the same seed-matched demand trajectories, the same per-period budget B and capacity ceiling V, and the same proportional down-scaling safety net applied immediately before execution. The columns record where the policies legitimately differ: the knob available for setting service level, and whether any feasibility mechanism acts before submission.

Policy	Service-level knob	Feasibility mechanism before submission
BL1 (ROP–EOQ)	Safety factor z, fixed at 1.645	None
BL2 (ML + Static)	Fixed days-of-supply rule	None
BL3 (ML + Adaptive)	z on rolling residual $\hat{\sigma}_{i,t}$, fixed at 1.645	None
BL4 (Per-SKU DQN)	Reward weights, held at defaults	None
BL5 (MAPPO)	Reward weights, held at defaults	None
BL6 (MAPPO + projection)	Reward weights, held at defaults	$\Pi_{\text{bud}}, \Pi_{\text{cap}}$ applied post hoc
BL7 (LLM-Agent)	Prompt-level service target	None
AAIRM	Stockout-penalty weight w_p, swept on the cost–service frontier	Full C5 operator chain, inside the training loop
